# Force sharing and force generation by two teams of elastically coupled molecular motors

**DOI:** 10.1038/s41598-018-37126-0

**Published:** 2019-01-24

**Authors:** Mehmet Can Uçar, Reinhard Lipowsky

**Affiliations:** Theory and Bio-Systems, Max Planck Institute of Colloids and Interfaces, Science Park Golm, 14424 Potsdam, Germany

## Abstract

Many active cellular processes such as long-distance cargo transport, spindle organization, as well as flagellar and ciliary beating are driven by molecular motors. These motor proteins act collectively and typically work in small teams. One particularly interesting example is two teams of antagonistic motors that pull a common cargo into opposite directions, thereby generating mutual interaction forces. Important issues regarding such multiple motor systems are whether or not motors from the same team share their load equally, and how the collectively generated forces depend on the single motor properties. Here we address these questions by introducing a stochastic model for cargo transport by an arbitrary number of elastically coupled molecular motors. We determine the state space of this motor system and show that this space has a rather complex and nested structure, consisting of multiple activity states and a large number of elastic substates, even for the relatively small system of two identical motors working against one antagonistic motor. We focus on this latter case because it represents the simplest tug-of-war that involves force sharing between motors from the same team. We show that the most likely motor configuration is characterized by equal force sharing between identical motors and that the most likely separation of these motors corresponds to a single motor step. These likelihoods apply to different types of motors and to different elastic force potentials acting between the motors. Furthermore, these features are observed both in the steady state and during the initial build-up of elastic strains. The latter build-up is non-monotonic and exhibits a maximum at intermediate times, a striking consequence of mutual unbinding of the elastically coupled motors. Mutual strain-induced unbinding also reduces the magnitude of the collectively generated forces. Our computational approach is quite general and can be extended to other motor systems such as motor teams working against an optical trap or mixed teams of motors with different single motor properties.

## Introduction

Within the living cell, cytoskeletal motors drive many essential processes such as the organization of the mitotic spindle, the powering of flagella and cilia, and the long-distance transport of membrane-bound organelles, neuronal vesicles, or mRNAs. Two ubiquitous motor species found in eukaryotic cells are conventional kinesin (or kinesin-1) and cytoplasmic dynein which have opposite polarities, walking preferentially towards the plus and minus ends of microtubules, respectively^1,2^. These motor proteins frequently work in teams of multiple motors^3–6^, which may lead to a tug-of-war between several dyneins and one or a few kinesins^3,7–9^. As a team, the motors can collectively generate large forces^8^, *e.g*., to organize mitotic spindles^10,11^ or to pull nanotubes from cellular membranes^12,13^. However, both theoretically and experimentally, the underlying mechanism of force generation and force sharing by several motors remains controversial^14–17^.

**Figure 1:**
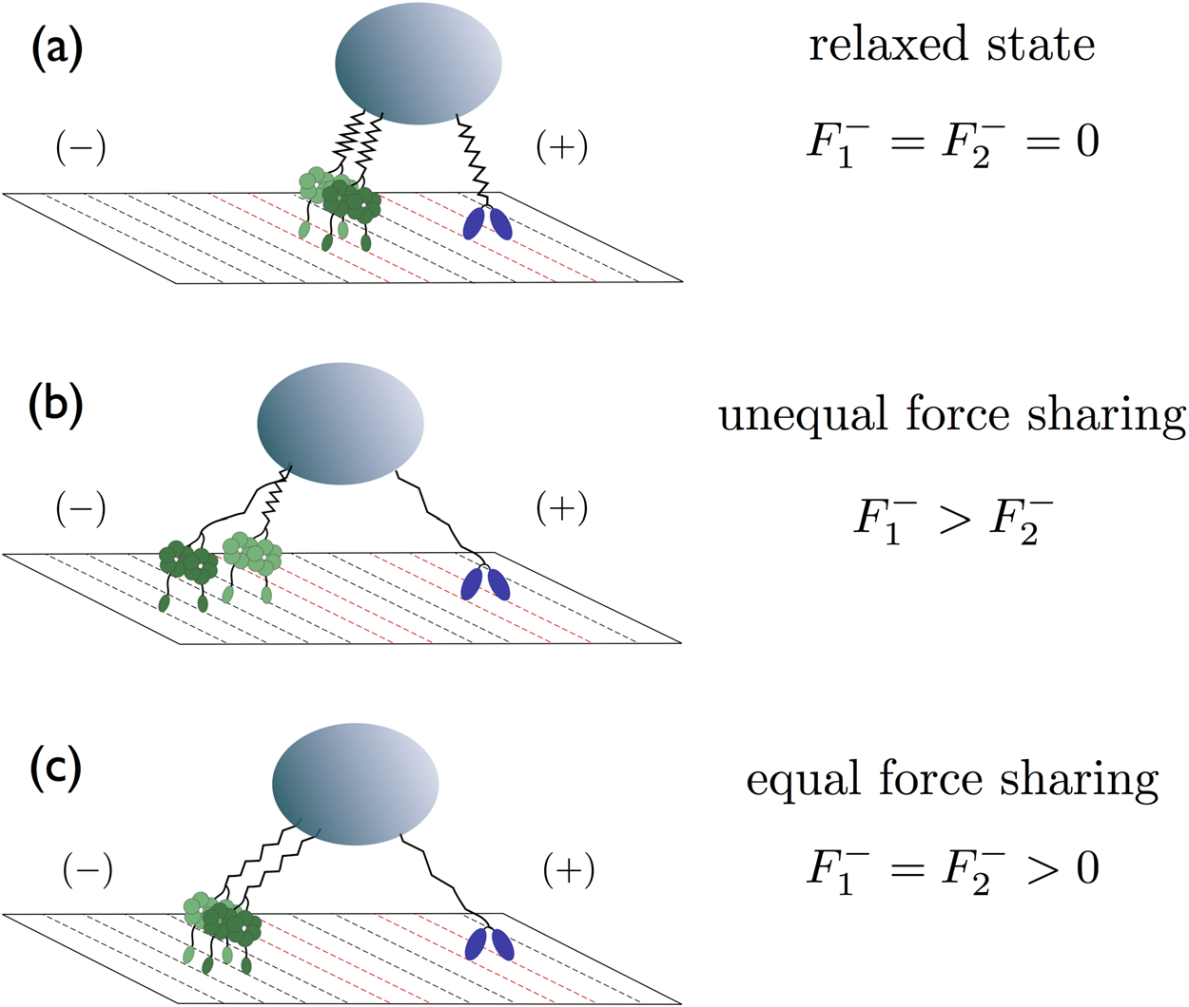
Different force sharing configurations of molecular motors engaged in a tug-of-war: Three elastic states of two minus-end directed dyneins (green) and one plus-end directed kinesin (blue) pulling on the same cargo (gray). The two dyneins experience the load forces ${F}_{1}^{-}$ and ${F}_{2}^{-}$: (**a**) All elastic linkers are relaxed and the two dyneins do not experience any force; (**b**) The elastic linker of the leftmost dynein is stretched more strongly than the linker of the other dynein, leading to different forces, *i.e*., ${F}_{1}^{-} > {F}_{2}^{-}$, and, thus, to unequal sharing of the force generated by the kinesin; and (**c**) The elastic linkers of the two dyneins experience the same stretching, corresponding to equal force sharing.

In this paper, we address these issues, focusing on motors that are elastically coupled to a common cargo. The different configurations of such a motor-cargo system are illustrated in Fig. 1 for the case of three motors, two dyneins and one kinesin. The relaxed state in which the elastic linkers do not generate any motor-motor forces is displayed in Fig. 1a. After one or a few steps of the individual motors, this system will attain motor-cargo configurations with unequal force sharing as in Fig. 1b or with equal force sharing as in Fig. 1c.

Force sharing is intimately related to collective force generation. If equal force sharing as in Fig. 1c were the typical configuration and if the motors remained bound to the filament, one might expect that a motor team with *N* motors can generate an overall force of approximately *NF*_*s*_, where *F*_*s*_ is the stall force of a single motor, *i.e*., that the collectively generated force increases additively with the size of the motor team. On the other hand, if unequal force sharing as represented in Fig. 1b were the typical situation, the leading motor in a motor team would bear most of the load, and a motor team with *N* motors could only generate forces that are significantly smaller than *NF*_*s*_.

An additive force generation mechanism based on equal force sharing was theoretically predicted in previous studies^4,5^ and is consistent with the force measurements from several experiments^7,8,10,18,19^, but seems to disagree with those from Jamison *et al*.^20^ and Furuta *et al*.^21^. In the latter studies, the overall force generated by several kinesin motors onto a cargo was observed to increase sub-additively with the motor number. This observation was taken to provide evidence for unequal force sharing^17^. Furthermore, several simulation studies of a tug-of-war between elastically coupled motors that undergo discrete mechanical steps also emphasized unequal force sharing without an explicit analysis of the underlying motor-cargo configurations^22–24^. One mechanism proposed in the literature^8,25,26^ for the low cooperativity among kinesin motors is based on the view that the typical motor configurations involve one leading motor that mainly carries the load. However, whether these unequal force sharing configurations dominate over the configurations with equal force sharing has not been investigated in these studies.

Here we introduce a general theoretical framework for elastically coupled motors and show that the elastic coupling between individual motors introduces a rather complex structure to the state space of such a system. We thus focus on the simplest nontrivial case of two identical motors pulling a common cargo against one antagonistic motor as in Fig. 1. We show that the most likely configuration of the steady state is characterized by equal force sharing between motors of the same team, regardless of the motor type. In fact, equal force sharing applies as well to the time-dependent build-up of strain forces arising from single motor steps. These features are quite general: they are also valid for motor teams with different stiffnesses and for motors that are elastically coupled by cable-like linkers. Furthermore, we calculate the average elastic forces arising in these systems and find that these forces are significantly smaller than those predicted by the non-elastic model^5^ which is based on equal force sharing. We show that this reduction of the collectively generated forces is caused by the unbinding of the motors from the filament during elastic strain generation. Our results demonstrate that collective force generation can be sub-additive *even though* motors of the same polarity share the force equally.

## Results

### Activity states, elastic substates, and elastic strain forces

We consider cargo transport by several elastically coupled motors which may bind to and unbind from the filament. In its bound state, each motor is described as a stochastic stepper with a certain step size $\ell$, using the motor parameters as deduced from single molecule experiments. The elastic linkers between the motors and the cargo are taken to be identical harmonic springs with spring constant *κ*. The case of two motor teams with different spring constants *κ*_−_ and *κ*_+_ is considered in sections *S1 Theoretical description of elastically coupled motors* and *S3* *Motor teams with different spring constants* in the *Supplementary Information* (SI). A detailed description of the single motor properties used in our study is given in the *Model and methods* section below.

### Activity states

A motor-cargo system consisting of a total number of *N*_−_ minus- and *N*_+_ plus-directed motors can attain different activity states (*n*_−_, *n*_+_) in which *n*_−_ minus and *n*_+_ plus motors are simultaneously bound to the filament and pull on the cargo. For two dyneins (*N*_−_ = 2) in a tug-of-war against a single kinesin motor (*N*_+_ = 1), for example, as depicted in Fig. 1, one needs to consider the activity state (2, 1) in which all three motors are bound to the filament, the state (2, 0) with two active dyneins^27^, the state (1, 1) with one active dynein and one active kinesin^28^, the states (1, 0) and (0, 1) corresponding to only one active motor, and the diffusive cargo state (0,0) with no active motors. We will consider motors from the same team to be distinguishable even if they are identical in the sense that they are built up from identical peptide chains. Experimentally, one can label the two identical motors separately using, *e.g*., different fluorescent dyes^29^ or quantum dots^30^ for each motor. Therefore, in the case of two dyneins against one kinesin, we have to distinguish two different (1, 1) and (1, 0) activity states, depending on which dynein is bound to the filament. We will denote these different states by (1, 0)_1_ and (1, 0)_2_, as well as (1, 1)_1_ and (1, 1)_2_, where the subscripts 1 and 2 correspond to dynein 1 and dynein 2, respectively. Alternatively, we could label the activity states by 3-tuples (*m*_1_,*m*_2_|*p*_1_) with occupation numbers *m*_*i*_ = 0, 1 for the two minus motors and *p*_1_ = 0,1 for the plus motor. The latter notation is more cumbersome but can be directly extended to the general case *via* (*n*_−_ + *n*_+_)-tuples $({m}_{1},\ldots ,{m}_{{n}_{-}}|{p}_{1},\ldots ,{p}_{{n}_{+}})$ with occupation numbers *m*_*i*_ = 0,1 and *p*_*j*_ = 0,1.

**Figure 2:**
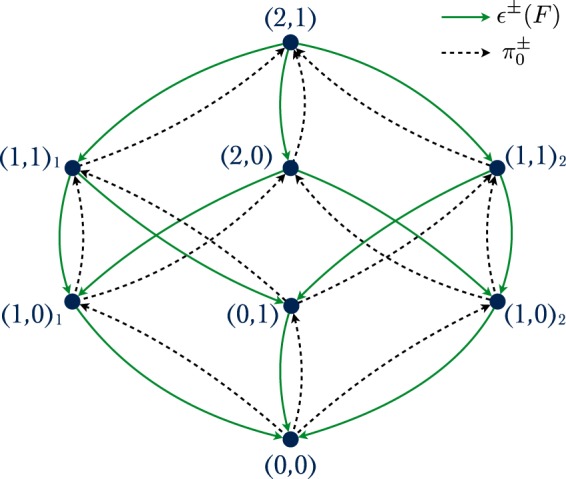
Activity states for cargo transport by *N*_−_ = 2 dynein and *N*_+_ = 1 kinesin motors**:** Network of eight different activity states (*n*_−_, *n*_+_) with *n*_−_ minus and *n*_+_ plus motors simultaneously bound to the filament. The two dyneins are distinguished by subscripts 1 and 2: Dynein 1 is bound to the filament in the activity states (1, 1)_1_ and (1, 0)_1_ while dynein 2 is bound for (1, 1)_2_ and (1, 0)_2_. Transitions between different activity states are governed by the binding and unbinding rates ${\pi }_{0}^{\pm }$ (broken lines) and *ε*^±^(*F*) (solid lines), respectively.

For *N*_−_ = 2 and *N*_+_ = 1, a coarse-grained representation of the state space in terms of the activity states alone is depicted in Fig. 2. In this representation, the eight activity states (*n*_−_, *n*_+_) = (2,1), (2, 0), (1, 1)_1_, (1, 1)_2_, (1, 0)_1_,(1, 0)_2_, (0, 1), and (0, 0) are connected *via* motor unbinding and rebinding transitions with rates *ε*^±^(*F*) and ${\pi }_{0}^{\pm }$, respectively, for details see *Model and methods* below, and the SI. In general, a system consisting of one motor team with *N*_−_ distinguishable minus motors and another motor team with *N*_+_ distinguishable plus motors can attain ${2}^{{N}_{-}+{N}_{+}}$ different activity states.

### Elastic substates

**Figure 3:**
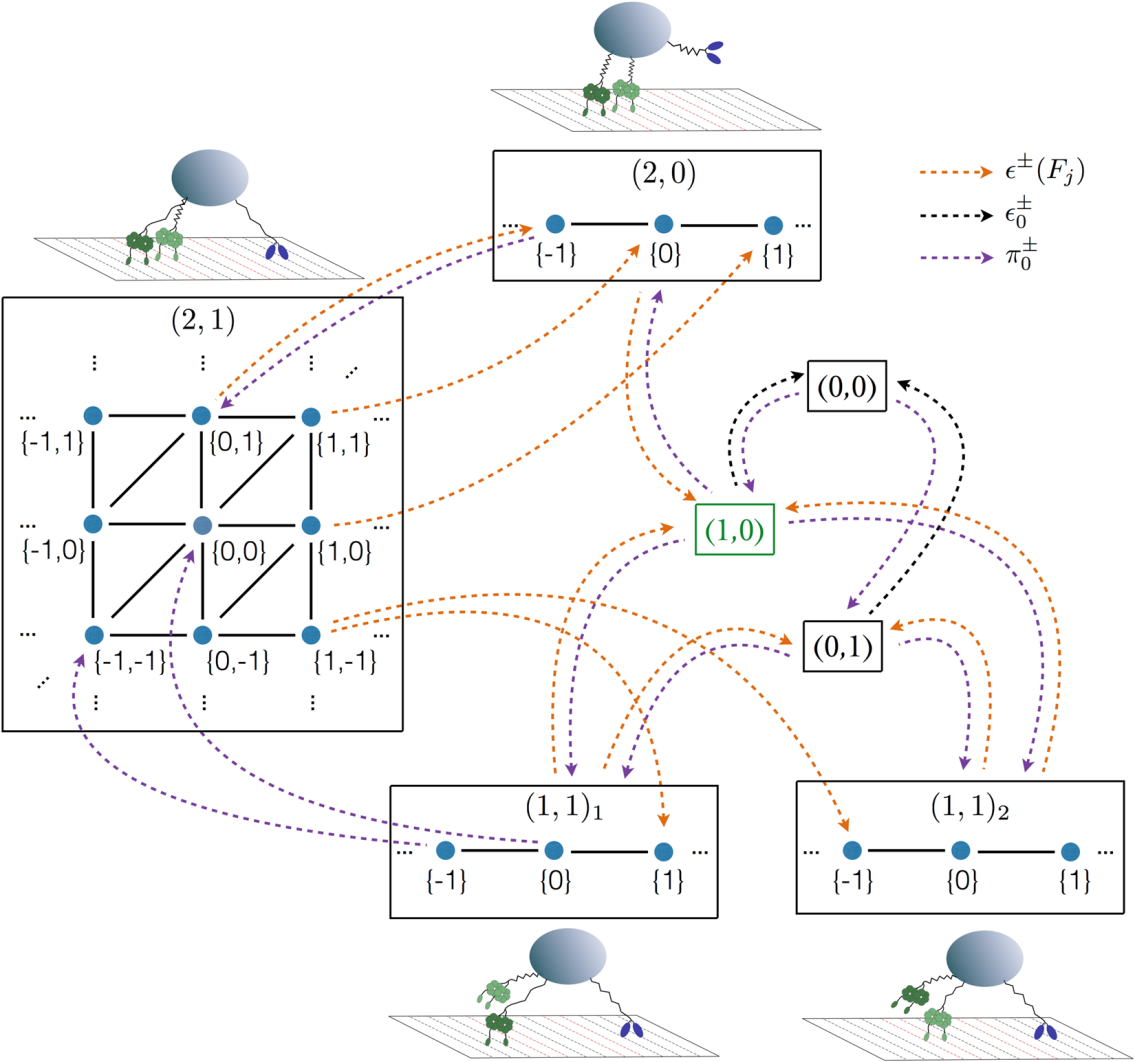
Complete state space for cargo transport by *N*_−_ = 2 dynein and *N*_+_ = 1 kinesin motors: Eight activity states (*n*_−_, *n*_+_) as in Fig. 2 with their elastic substates in curly brackets. The state (1, 0) combines the two activity states (1, 0)_1_ and (1, 0)_2_, compare Fig. 2. Transitions between elastic substates within a single activity state (*n*_−_, *n*_+_) are depicted by solid lines (black) and governed by the forward and backward stepping rates of the motors. For visual clarity, only a few transitions (broken lines, colored) between elastic substates of different activity states are shown explicitly. The unbinding rate of each active motor depends on the elastic strain experienced by the motor and thus on the elastic substate, see SI for details.

When one of the bound motors performs a forward or backward step, it changes the motor-motor separations along the filament and, thus, the elastic forces experienced by the motors. As a consequence, motor configurations with different motor-motor separations define different elastic substates of each activity state with more than a single motor attached to the filament, *i.e*., with *n*_−_ + *n*_+_ ≥ 2, as shown in Fig. 3. In each substate, different elastic forces are experienced by the individual motors, and thus different force-dependent rates govern the transitions between these substates. As an example, let us consider a cargo that dwells in the activity state (*n*_−_, *n*_+_) = (2, 1) with two minus-end directed and one plus-end directed motor. The forces experienced by the motors and the cargo depend on the two motor-motor separations between the kinesin and each of the two dyneins. As a consequence, the elastic substates of the activity state (2, 1) form a two-dimensional lattice as shown in the leftmost box of Fig. 3. As long as all three motors remain attached to the filament, single motor steps lead to transitions between neighboring sites of this lattice. However, when dynein 1 or dynein 2 unbinds from the filament, we leave the activity state (2, 1) towards the activity states (1, 1)_2_ and (1, 1)_1_, respectively, see the two lower boxes in Fig. 3. Each of these latter activity states consists of a 1-dimensional lattice of elastic substates, corresponding to the different possible separations of the bound dynein and the bound kinesin. Likewise, when the kinesin motor unbinds from the filament, we leave the activity state (2, 1) and enter the activity state (2, 0), which consists of a 1-dimensional lattice of elastic substates, corresponding to the different possible separations of the two dyneins, see upper box in Fig. 3.

### Force balance

Newton’s third law implies that the sum of all elastic forces acting on the cargo must vanish. This force balance condition can be used to express the cargo position in terms of the positions of all active motors. As a result, one finds that a single step of an individual motor changes the position of the cargo in the motor’s stepping direction by $\ell /({n}_{-}+{n}_{+})$, as derived in *S1 Theoretical description of elastically coupled motors* in the SI. In a motor-cargo system with three active motors, for instance, a forward step stretches the elastic linker of the stepping motor by $\ell -\ell \mathrm{/3}=2\ell \mathrm{/3}$, increasing the elastic force acting on the motor by $2\kappa \ell \mathrm{/3}$ as follows from Hooke’s law. Because of the cargo shift, the linkers of the remaining motors of the same team will then be compressed by $\ell \mathrm{/3}$ whereas the linkers of the motors in the opposing team will be stretched by the same amount. For simplicity, we will now focus on the activity state (2,1) as illustrated in Fig. 1, and consider the general case in the SI.

### Tug-of-war between two dyneins and one kinesin

We label the two dyneins by the indices *j* = 1, 2 and the single kinesin by *j* = 3. The elastic linker connecting the motor *j* to the cargo has a certain rest length ${L}_{\parallel }$, and the deviation of the linker length *L*_*j*_ from its rest length defines the elastic displacement ${u}_{j}\equiv {L}_{j}-{L}_{\parallel }$. For motor teams with identical spring constants, any elastic substate of the activity state (2, 1) is then characterized by the three-dimensional displacement vector **u** = (*u*_1_, *u*_2_, *u*_3_) with *u*_1_ + *u*_2_ = *u*_3_ as follows from the force balance condition. Starting from a relaxed state with **u**_0_ = (0, 0, 0) as in Fig. 1a, one can then reach all possible elastic substates by successive mechanical steps of the three motors. In general, ${L}_{\parallel }$ and $\ell$ are two independent length scales and need not be commensurate. The latter case introduces another parameter that we have eliminated here for the sake of clarity. We parametrize the elastic substates by a three-dimensional lattice with sites {*s*_1_, *s*_2_, *s*_3_} and integer-valued *s*_*j*_. Positive values of *s*_*j*_ represent *s*_*j*_ successive forward steps, negative values of *s*_*j*_ correspond to |*s*_*j*_| successive backward steps. The associated displacement vector *u* has the form

1 $${\bf{u}}\{{s}_{1},{s}_{2},{s}_{3}\}={{\bf{u}}}_{0}+\ell \sum _{j\mathrm{=1}}^{3}{s}_{j}{{\bf{b}}}_{j}\,,$$

with the two basis vectors

2 $${{\bf{b}}}_{1}\equiv (\displaystyle \frac{2}{3},-\displaystyle \frac{1}{3},\displaystyle \frac{1}{3})\,{\rm{and}}\,{{\bf{b}}}_{2}\equiv (-\displaystyle \frac{1}{3},\displaystyle \frac{2}{3},\displaystyle \frac{1}{3})\,,$$

as well as the linearly dependent vector

3 $${{\bf{b}}}_{3}\equiv (\tfrac{1}{3},\tfrac{1}{3},\tfrac{2}{3})={{\bf{b}}}_{1}+{{\bf{b}}}_{2}.$$

Each vector **b**_*j*_ corresponds to a single forward step of the individual motor labeled by *j*. The relation in equation (3) reflects the force balance condition and implies that the displacement vectors have the general form

4 $${\bf{u}}\{{r}_{1},{r}_{2}\}={{\bf{u}}}_{0}+\ell \,[{r}_{1}{{\bf{b}}}_{1}+{r}_{2}{{\bf{b}}}_{2}]\,,\,{\rm{with}}\,{r}_{j}\equiv {s}_{j}+{s}_{3}$$

for *j* = 1, 2, *i.e*., all elastic substates are located in a 2-dimensional plane generated by the two basis vectors **b**_1_ and **b**_2_. Furthermore, the elastic forces *F*_*j*_ acting on the three motors in substate {*r*_1_, *r*_2_} = {*s*_1_ + *s*_3_, *s*_2_ + *s*_3_} are given by the force vector

5 $${\bf{F}}\{{r}_{1},{r}_{2}\}=({F}_{1},{F}_{2},{F}_{3})=\kappa \,{\bf{u}}\{{r}_{1},{r}_{2}\},$$

with *F*_1_ + *F*_2_ = *F*_3_ due to the force balance condition. For a given set of motor parameters, equations (4) and (5) fully determine the strain-dependent single motor rates of all motors in each elastic substate {*r*_1_, *r*_2_}, for details see SI. The same description can be used to study the tug-of-war between two kinesins and one dynein by defining the motors labelled by *j* = 1, 2 with kinesin properties and the single opposing motor with dynein properties. In this study, we examine two different types of dynein motors; “strong” and “weak” dynein, which correspond to yeast and mammalian cells, respectively. In accordance with several experimental findings^3,8,31–34^, we model yeast dynein as a slow motor with a high stall force, and mammalian dynein as a fast motor with a low stall force value, see Table 1 for the motor parameters used here.

**Table 1:** Single motor parameters used here.

Parameter	kinesin-1	dynein_S_	dynein_W_
Unbinding rate *ε*_0_ [s^−1^]	0.66^50^	1*	1^51^
Binding rate *π*_0_ [*s*^−1^]	5^13^	5*	5*
Stall force *F*_*s*_ [pN]	7^49^	7^31,52^	1.1^8,33^
Detachment force *F*_*d*_ [pN]	2.1^50^	2.9^43^	2.9^43^
Stiffness *κ* [pN/nm]	0.2^53^	0.2*	0.2*
Step size $\ell$ [nm]	8^49,53^	8^32^	8^52^
Step ratio *q*_0_	800^49^	4^32^	4*
Velocity *v*_0_ [nm/s]	740^50^	85^32^	800^52^
Min. velocity *v*_min_ [nm/s]	89^†^	10^†^	96^†^
Max. velocity *v*_max_ [nm/s]	829^†^	95^†^	896^†^

The first column for kinesin-1 includes the motor parameters that have been recently measured by Andreasson *et al*.^50^. The values for “strong” and “weak” dynein (columns dynein_S_ and dynein_W_) correspond to yeast and mammalian cells, respectively. An asterisk superscript indicates a parameter for which we did not find experimental data in the literature; the corresponding parameter value was set equal to the experimentally deduced value of another type of motor. Velocity values depicted by the dagger superscript are estimated by *v*_min_ ≃ 0.12*v*_0_ and *v*_max_ ≃ 1.12*v*_0_, which represent the limiting values for large superstall forces and for large assisting forces, respectively, and provide a convenient parametrization^27^ of the force-velocity relations.

### Steady state for tug-of-war between three motors

We focus on the case of two identical motors against a single opposing motor because this choice provides the simplest nontrivial example for which one can study force sharing between identical motors in a systematic manner. We represent the complete network of activity states by a transition rate matrix and calculate the steady state probability distribution over the complete network for different choices of motor parameters, for details see SI.

We distinguish the steady state probabilities *P*^st^(*n*_−_, *n*_+_) for the activity states (*n*_−_, *n*_+_) of the cargo from the steady state probabilities *p*^st^{*r*_1_, *r*_2_} to find the motor-cargo system in the elastic substates {*r*_1_, *r*_2_} of the activity state (2,1) with the normalization conditions ${\sum }_{{r}_{1},{r}_{2}}{p}^{{\rm{st}}}\{{r}_{1},{r}_{2}\}={P}^{{\rm{st}}}\mathrm{(2,1)}$ and ${\sum }_{{n}_{-},{n}_{+}}{P}^{{\rm{st}}}({n}_{-},{n}_{+})=1$. In addition, we define the conditional probabilities $\hat{p}$^st^{*r*_1_,*r*_2_}≡*p*^s*t*^{*r*_1_,*r*_2_}/*P*^st^(2,1) to find the system in the elastic substate {*r*_1_,*r*_2_} given that the cargo is in the activity state (2,1). Note that the numbers *r*_1_ and *r*_2_ determine the separation of the first and second motor of the same team from the single opposing motor such that the elastic substate with *r*_1_ = *r*_2_ = 0 represents the relaxed state of the system, whereas all configurations with *r*_1_ = *r*_2_ correspond to equal force sharing.

**Figure 4:**
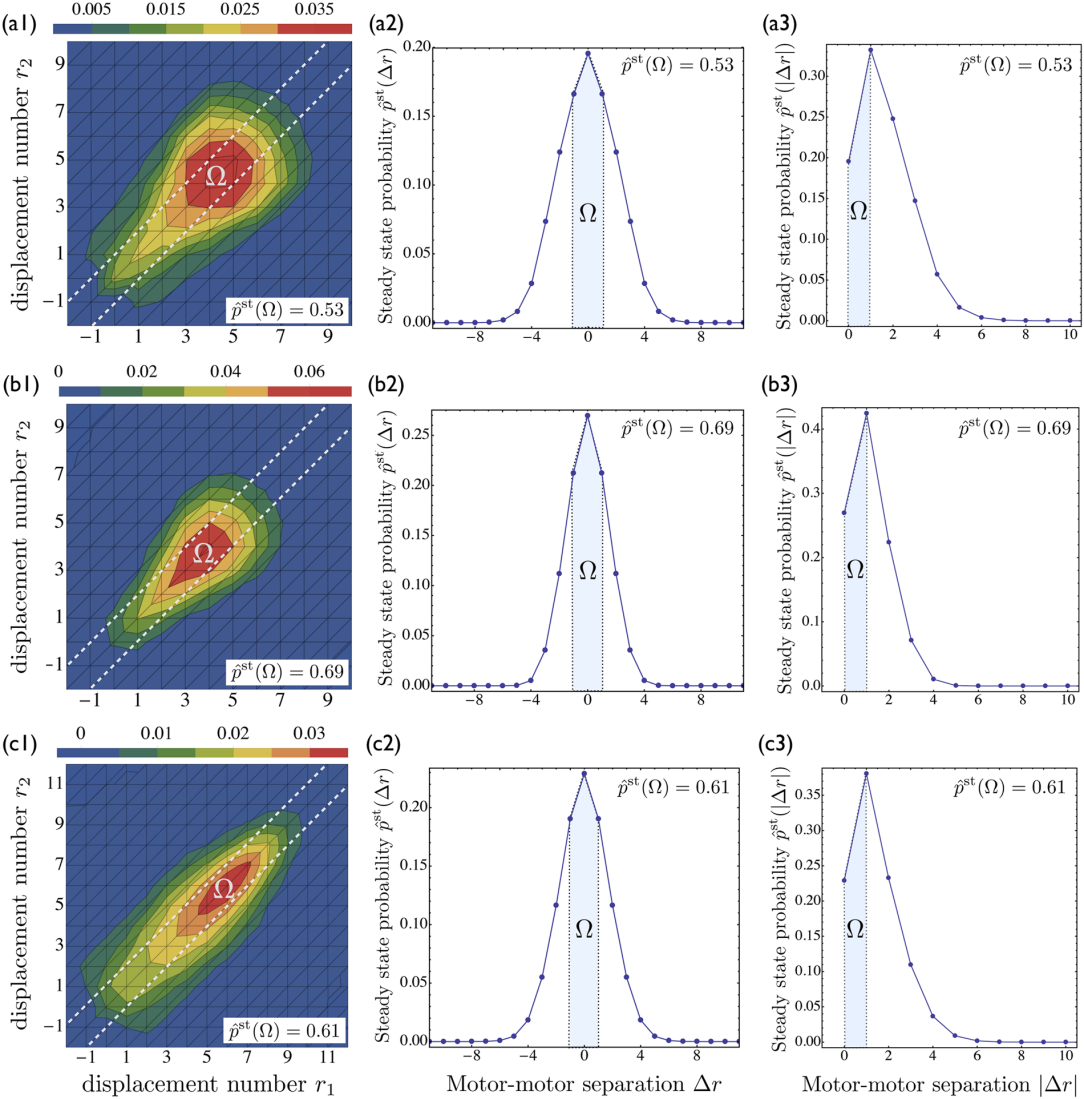
Force sharing between identical motors engaged in a tug-of-war: (**a**) One kinesin against two strong dyneins; (**b**) One kinesin against two weak dyneins; and (**c**) one strong dynein against two kinesins. (**a1**,**b1**,**c1**) Conditional steady state probabilities $\hat{p}$^st^{*r*_1_, *r*_2_} for the elastic substates {*r*_1_, *r*_2_} = {*s*_1_ + *s*_3_, *s*_2_ + *s*_3_}. The color code provides the normalized probabilities. Because these probability distributions exhibit a single maximum at the diagonal, the most likely motor-cargo configurations are characterized by equal force sharing. The regions Ω contain all motor configurations for which the separation of the two identical motors does not exceed a single motor step, corresponding to |Δ*r*| = |*r*_2_ − *r*_1_| ≤ 1. These regions are bounded by the two broken lines (white) and are visited with probabilities $\hat{p}$^st^(Ω) > 0.5 as given in the insets; (**a2,b2,c2**) Steady state probabilities for the separation Δ*r* of the two identical motors; and (**a3,b3,c3**) Steady state probabilities for the absolute value |Δ*r*| which reveal that the most likely motor-motor separation |Δ*r*| always corresponds to a single motor step. The separation Δ*r* is given in units of the step size $\ell =8\,$ nm.

The conditional probabilities $\hat{p}$^st^{*r*_1_, *r*_2_} are displayed in Fig. 4 for three different cases: (a) two strong (yeast) dyneins against one kinesin, (b) two weak (mammalian) dyneins against one kinesin, and (c) one strong dynein against two kinesins, see Table 1. All of these probabilities have a *single* maximum at the diagonal, *i.e*., the most likely configuration is one of equal force sharing in all three cases. The region Ω in the first column of Fig. 4 contains all motor configurations for which the absolute separation of the two dyneins or the two kinesins does not exceed a single motor step, *i.e*., |Δ*r*| = |*r*_2_ − *r*_1_| ≤ 1. The probability to find the system in this region satisfies $\hat{p}$^st^(Ω) > 0.5. Furthermore, the steady state distributions for the absolute values |Δ*r*| of the motor-motor separations as displayed in the third column of Fig. 4 reveal that the most likely separation between the two identical motors corresponds to a single motor step.

### Reduction of average force by elastic coupling

**Figure 5:**
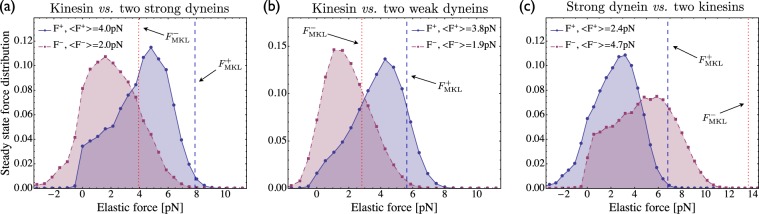
Steady state distributions for elastic forces generated by three-motor systems: In all cases, the two identical motors have the same force distribution and experience the same average force. The non-elastic MKL model^5^ predicts the forces ${F}_{{\rm{M}}{\rm{K}}{\rm{L}}}^{+}$ (dashed vertical line) and ${F}_{{\rm{M}}{\rm{K}}{\rm{L}}}^{-}$ (dotted vertical line) acting on the individual plus and minus motors, respectively. (**a**) For strong dyneins, we obtain the average elastic force 〈*F*^+^〉 = 4.0 pN compared to the cargo force ${F}_{{\rm{M}}{\rm{K}}{\rm{L}}}^{+}\mathrm{=7.9}$ pN; (**b**) for weak dyneins, 〈*F*^+^〉 = 3.8 pN compared to ${F}_{{\rm{M}}{\rm{K}}{\rm{L}}}^{+}\mathrm{=5.6}$ pN; (**c**) for kinesins, 〈*F*^−^〉 = 4.7 pN compared to ${F}_{{\rm{M}}{\rm{K}}{\rm{L}}}^{-}\mathrm{=13.6}$ pN. The average elastic forces are lower than the predictions of the non-elastic MKL model because the motors typically undergo strain-induced unbinding before they can generate large forces. The difference becomes larger if the motors are characterized by larger stall forces and larger unbinding rates.

The conditional probabilities $\hat{p}$^st^{*r*_1_, *r*_2_} for the elastic substates {*r*_1_, *r*_2_} as displayed in the first column of Fig. 4 determine the average elastic force 〈*F*_*j*_〉 acting on motor *j via* the first moments

6 $$\langle {F}_{j}\rangle =\kappa \ell \sum _{{r}_{1},{r}_{2}}{\hat{p}}^{{\rm{s}}{\rm{t}}}\{{r}_{1},{r}_{2}\}[{r}_{1}\,{b}_{\mathrm{1,}j}+{r}_{2}\,{b}_{\mathrm{2,}j}]\,,$$

where *b*_1,*j*_ and *b*_2,*j*_ are the *j*-components of the two basis vectors **b**_1_ and **b**_2_ as defined in equation (2). Note that each pair {*r*_1_, *r*_2_} of displacement numbers determines the elastic forces **F**{*r*_1_, *r*_2_} = (*F*_1_, *F*_2_, *F*_3_) acting on all three motors, as described by equation (5). The steady state probabilities plotted in the first column of Fig. 4 can thus be translated into steady state force distributions. If more than one elastic substate {*r*_1_, *r*_2_} leads to the same force value ${F}_{j}^{\ast }$ acting on the *j*-th motor, we sum over the corresponding probabilities to obtain the overall probability for this force value. The steady state distributions of the elastic forces acting on the individual motors are plotted in Fig. 5. For comparison, the predictions of the non-elastic model introduced by Müller, Klumpp, and Lipowsky (MKL)^5^ are also included: in Fig. 5a,b the single kinesin is exposed to the average force ${F}_{{\rm{M}}{\rm{K}}{\rm{L}}}^{+}\equiv {F}_{{\rm{c}}{\rm{a}}}$ whereas the two dyneins experience the same average force given by ${F}_{{\rm{M}}{\rm{K}}{\rm{L}}}^{-}\equiv {F}_{{\rm{c}}{\rm{a}}}\mathrm{/2=}{F}_{{\rm{M}}{\rm{K}}{\rm{L}}}^{+}\mathrm{/2}$, equally sharing the force *F*_ca_ experienced by the cargo^28^. In Fig. 5c, on the other hand, the cargo force is given by the force acting on the single dynein, *i.e*., ${F}_{{\rm{M}}{\rm{K}}{\rm{L}}}^{-}\equiv {F}_{{\rm{c}}{\rm{a}}}$, and the two kinesins share this force equally.

**Figure 6:**
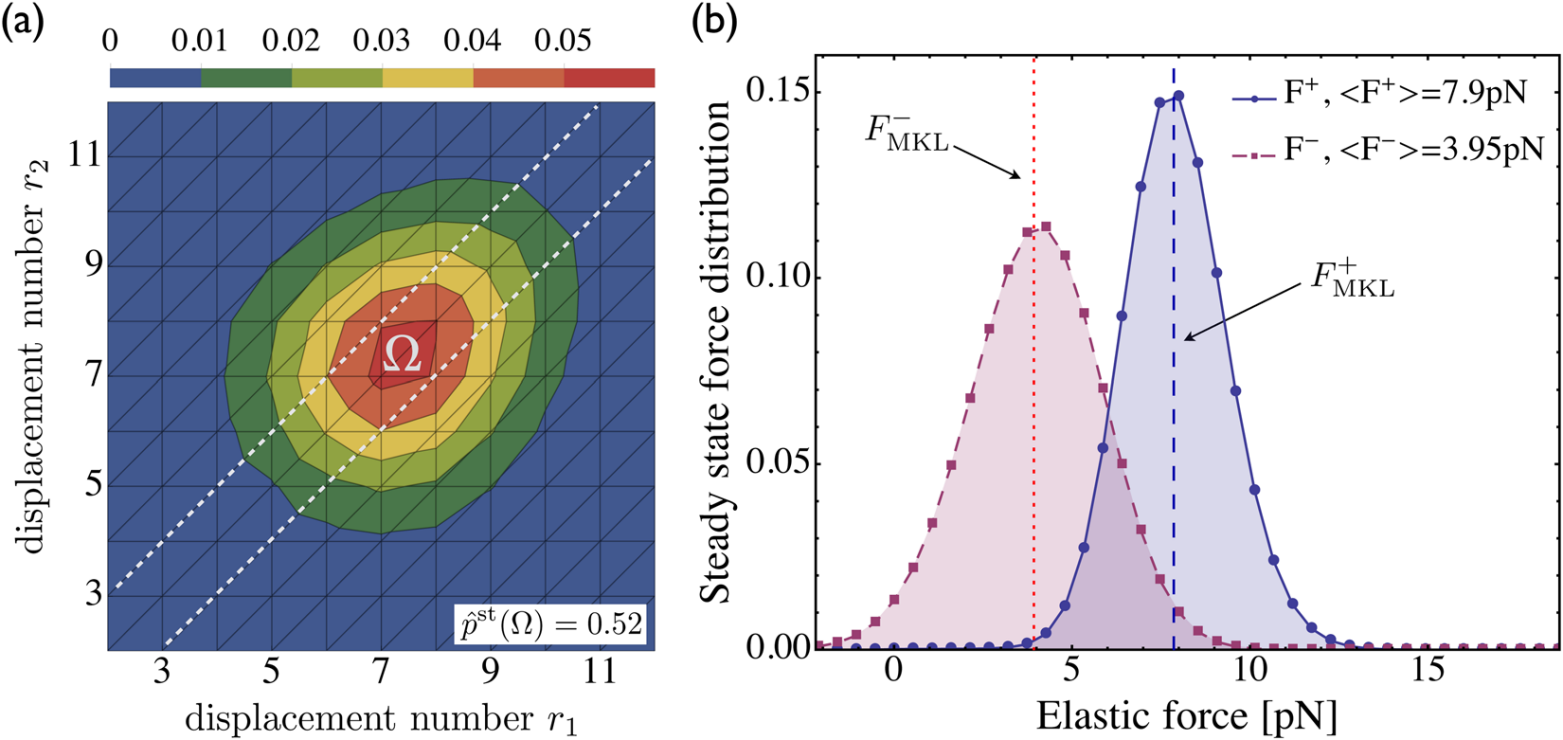
Limit of small unbinding rates for the tug-of-war between two strong dyneins and a single kinesin: (**a**) Steady state probability distribution $\hat{p}$^st^{*r*_1_,*r*_2_} for the elastic substates {*r*_1_, *r*_2_} of the (2, 1)-activity state in the limit of small unbinding rates as described by equation (10) with ${\varepsilon }_{0}^{+}={\varepsilon }_{0}^{-}\mathrm{=0.01}\,/$ s and ${F}_{d}^{+}={F}_{d}^{-}\mathrm{=6}\,$ pN. As in Fig. 4, the distribution again exhibits a single maximum on the diagonal; and (**b**) Steady state distributions of the elastic interaction forces obtained from the probability distribution plotted in (**a**). The solid curve describes the elastic forces acting on the single kinesin, as well as on the cargo, whereas the dashed curve corresponds to the force distributions for each dynein. Vertical dashed and dotted lines correspond to the forces predicted by the non-elastic MKL model. Comparison with Fig. 5a reveals that the average elastic forces approach the predictions of the MKL model for small unbinding rates.

The average elastic forces 〈*F*_*j*_〉 as given by equation (6) are smaller than the cargo force predicted by the MKL model. The deviation of the average elastic forces from the MKL predictions increases for larger forces acting on the cargo, *e.g*., in the tug-of-war between kinesin and strong dynein, see Fig. 5a,c. This reduction in the average forces arises from the strain-induced unbinding of the elastically coupled motors before they can generate forces close to the cargo force *F*_ca_. In fact, in the limit of small unbinding rates, the average elastic forces do approach the cargo force *F*_ca_ as shown in Fig. 6. In contrast to the asymmetric force distributions in Fig. 5, which reflect the frequent strain-induced unbinding events in the steady state, the force distributions in Fig. 6b, corresponding to rare unbinding events, are essentially symmetric and nearly Gaussian.

### Equal force sharing during initial build-up of strain

**Figure 7:**
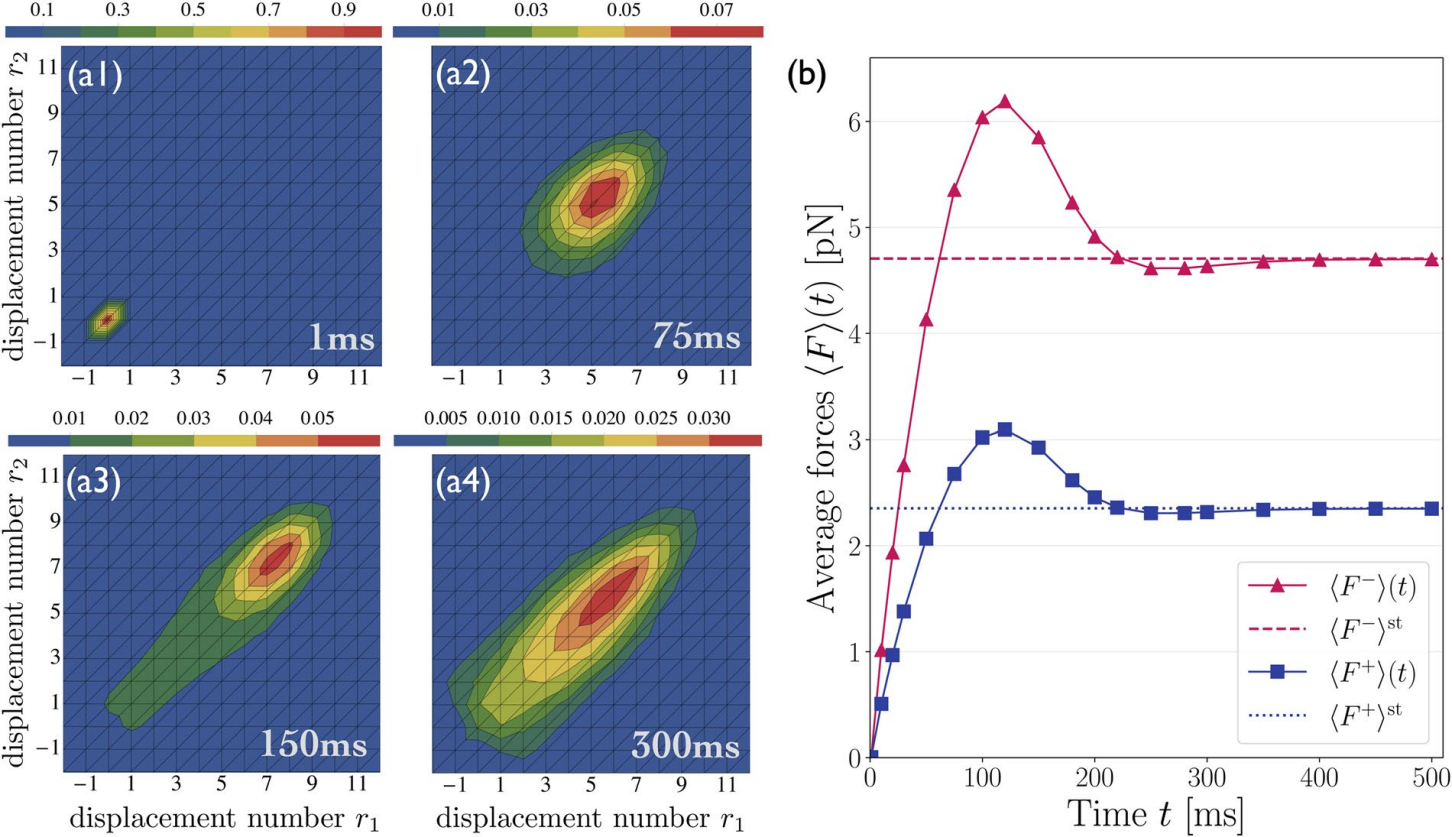
Initial build-up of elastic strain forces during the tug-of-war between two kinesins and one dynein: (**a1**–**a4**) Time evolution of the probability distribution $\hat{p}${*r*_1_, *r*_2_}(*t*) for the elastic states {*r*_1_, *r*_2_} of the tug-of-war between two kinesins and one strong dynein. The distribution reaches its steady state at $t\simeq 300$ ms. During the whole time evolution, the most likely configuration is characterized by equal force sharing between the two kinesins; and (**b**) Time evolution of the average elastic forces 〈*F*^±^〉(*t*) experienced by the dynein and kinesin motors, exhibiting maximal average forces 〈*F*^±^〉^max^ at $t\simeq 120$ ms. The elastic forces converge to their steady state values 〈*F*^−^〉^st^ (dashed horizontal line) and 〈*F*^+^〉^st^ (dotted horizontal line) for *t* ≥ 300 ms.

The distributions in Fig. 4 describe the long-time behavior of the elastic motor-cargo configurations, for which strain-induced unbinding events lead to a reduction of the average forces generated in the system. In principle, one could envisage that the initial build-up of elastic strain generation is dominated by configurations that correspond to unequal force sharing between motors from the same team. In order to test such a scenario, we investigate the time evolution of the probability distribution $\hat{p}\{{r}_{1},{r}_{2}\}$ over the elastic substates {*r*_1_, *r*_2_}, see SI for details. The time-dependent probability distributions $\hat{p}${*r*_1_, *r*_2_}(*t*) are plotted in Figs 7a1–a4 for the case of two kinesin motors in a tug-of-war against a single strong dynein. The motor-cargo system is taken to start at the relaxed state {*r*_1_ = 0, *r*_2_ = 0} at *t* = 0, and reaches its steady state after approximately *t* = 300 ms, see Fig. 7a4. Inspection of Figs 7a2 and a3 shows that the most likely configuration is also characterized by equal force sharing at the intermediate times *t* = 75 ms and *t* = 150 ms, *i.e*., before the system reaches its steady state.

The time evolution of the average elastic forces is displayed in Fig. 7b. The average elastic force 〈*F*^−^〉(*t*) acting on the single dynein motor is shared by the two kinesins equally, *i.e*., 〈*F*^+^〉(*t*) = 〈*F*^−^〉(*t*)/2. At approximately $t\simeq 120$ ms the forces reach a maximum, and relax to their steady state values 〈*F*^±^〉^st^ for *t* ≥ 300 ms. Recall that the MKL model predicts the cargo force ${F}_{{\rm{c}}{\rm{a}}}={F}_{{\rm{M}}{\rm{K}}{\rm{L}}}^{-}=13.6$ pN acting on the single dynein motor, see Fig. 5c. The maximal force that can be achieved during strain generation in the elastically coupled system, on the other hand, is only about ${\langle {F}^{-}\rangle }^{{\rm{\max }}}\simeq 6.2$ pN because of strain-induced unbinding. Thus, we see once more the importance of strain-induced unbinding: Kinesin and dynein motors initially stretch the elastic linkers to a large extent by taking successive steps, but strain-induced unbinding events eventually cause the motors to attain more relaxed linkers. If we suppress strain-induced unbinding by considering the limit of small unbinding rates, the average elastic forces 〈*F*^+^〉(*t*) and 〈*F*^−^〉(*t*) no longer exhibit maxima at intermediate times but increase monotonically with time as shown in *S4 Tug-of-war in the limit of small unbinding rates* in the SI. In the latter case, the long-time limit of the average forces is provided by the average forces of the non-elastic model.

### Two kinesins and one dynein with cable-like linkers

The results presented in Figs 4–7 were obtained for a model in which the motor linkers are described as harmonic springs, *i.e*., the motors respond to both stretching and compression forces. However, in a previous experimental study^35^, kinesin’s stiffness was observed to be an order of magnitude lower for compression than for stretching. It is thus instructive to consider nonharmonic cable-like motor linkers which behave like harmonic springs when stretched, but do not generate any forces when compressed, as assumed in several other studies^23,24,36^. As in the previous subsection, we will again focus on the tug-of-war between two kinesins and one strong dynein.

For cable-like linkers, the elastic force *F*_*j*_ acting on the *j*-th motor vanishes for $|{L}_{j}| < |{L}_{\parallel }|$, *i.e*., when the motor linker *L*_*j*_ is shorter than its rest length ${L}_{\parallel }$. The rest length ${L}_{\parallel }$ therefore becomes an essential parameter that determines the range of elastic substates of the complete state space, see Fig. 3, in which the motors step on the filament in a load-free manner. Kinesin’s rest length is primarily determined by its tail domain which leads to ${L}_{\parallel }\simeq 80-110$ nm^37,38^, and to ${L}_{\parallel }/\ell \simeq 10-13$. The latter ratio determines the fraction of elastic substates for which the motors do not experience any force and increases the number of available rebinding states for detached motors compared to the motor system with harmonic linkers. In our calculations, we took the rest length ${L}_{\parallel }$ to be 80 nm.

**Figure 8:**
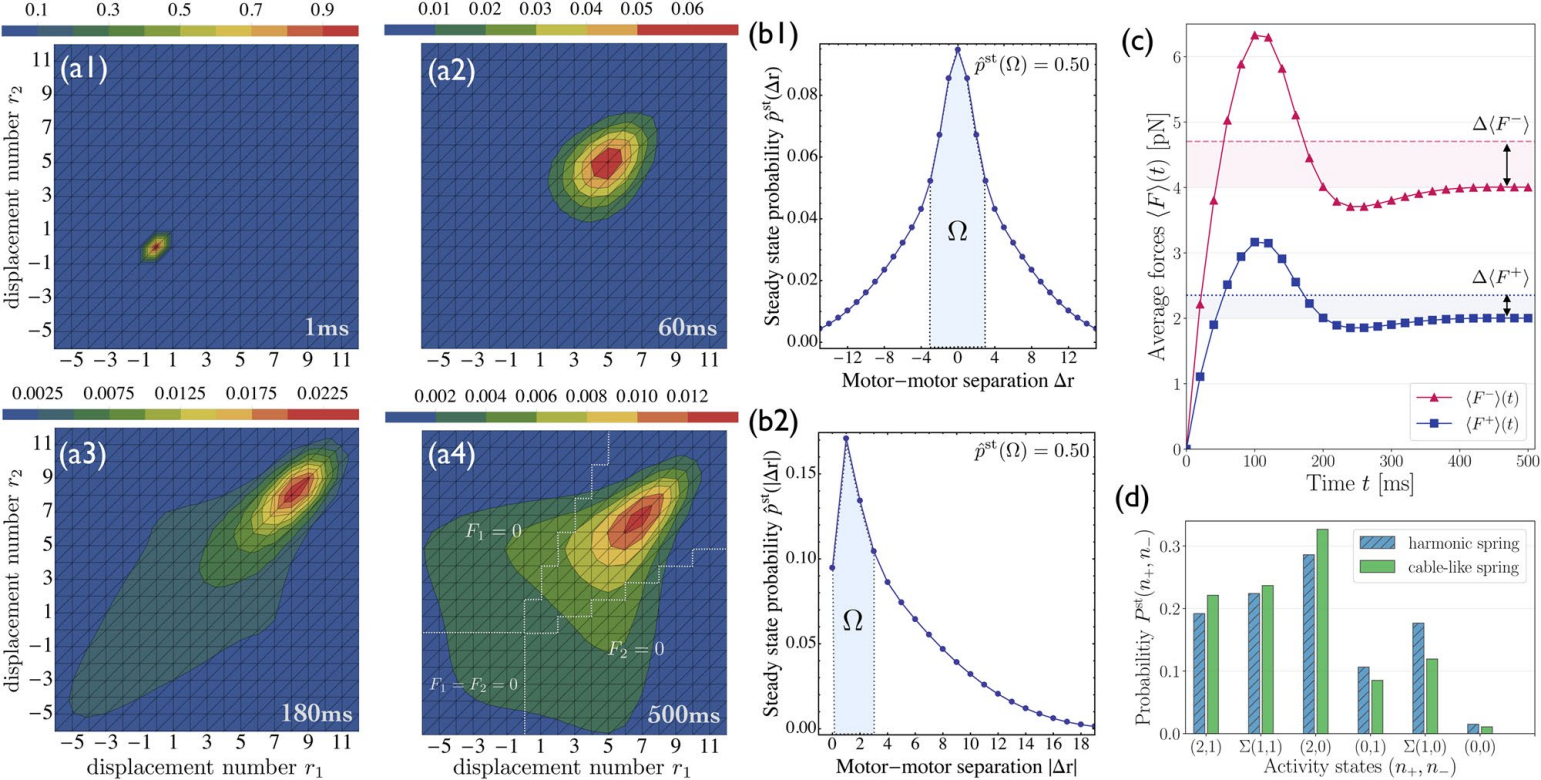
Two kinesins and one strong dynein elastically coupled by cable-like linkers: (**a1**–**a4**) Time evolution of the probability distribution $\hat{p}${*r*_1_, *r*_2_}(*t*) for the elastic states {*r*_1_, *r*_2_}. The distribution in (**a4**) as obtained after 500 ms is close to the steady state and provides non-zero probabilities for many off-diagonal states with *r*_1_ ≠ *r*_2_. The three regions separated by the white dotted lines in (**a4**) correspond to states where at least one of the kinesins can move in a load-free manner corresponding to *F*_1_ = 0 and/or *F*_2_ = 0; (**b1**,**b2**) Steady state probabilities ${\hat{p}}^{{\rm{s}}{\rm{t}}}${*r*_1_, *r*_2_} for the motor-motor separation Δ*r* and for its absolute value |Δ*r*|, which demonstrate that the most likely configuration is characterized by equal force sharing between the two kinesins, and the most likely separation is given by a single motor step; (**c**) The average elastic forces 〈*F*^±^〉(*t*) display a pronounced maximum at $t\simeq 100\,$ ms, and converge to the steady state values for *t* ≥ 300 ms. These timescales for maximal force generation and force relaxation are similar for both types of linkers, see Fig. 7b. The steady state force values, on the other hand, are somewhat smaller for the cable-like linkers than for the harmonic linkers, as indicated by the shaded regions and the corresponding differences Δ〈*F*^±^〉; and (**d**) Steady state probabilities *P*^st^(*n*_+_, *n*_−_) for the different activity states indicate that the overall cargo transport is largely unaffected by the different types of linkers.

The tug-of-war between two kinesins and one dynein, which are elastically coupled by cable-like linkers, leads to the time evolution of the conditional probability distribution $\hat{p}${*r*_1_, *r*_2_}(*t*) as displayed in Fig. 8a1–a4. The steady state is reached after approximately $t\simeq 300\,$ ms and leads to the distribution in Fig. 8a4. Inspection of this latter distribution shows that many off-diagonal states with *r*_1_ ≠ *r*_2_ have non-zero probabilities, indicating that unequal force sharing configurations have an increased overall probability compared to harmonic linkers as displayed in Fig. 4c1. This feature arises from the increased number of available substates {*r*_1_, *r*_2_} to which the detached motors can rebind with a relaxed linker, see the different regions with *F*_1_ = 0 and/or *F*_2_ = 0 in Fig. 8a4, separated by the white dotted lines. However, the most likely configuration for cable-like linkers is again characterized by equal force sharing, and the most likely separation between the two kinesins is again given by a single motor step, as shown in Fig. 8b1–b2.

As in the case of harmonic linkers, the initial build-up of mutual interaction forces exhibits a maximal force of about 6.3 pN and relaxes to the steady-state value for *t* ≥ 300 ms, see Fig. 8c. Interestingly, cable-like linkers generate about the same maximal force as harmonic linkers and need about the same time for force generation and force relaxation. The steady state force values are however somewhat smaller for the cable-like linkers than for the harmonic linkers, as indicated by the shaded regions in Fig. 8c and corresponding differences Δ〈*F*^±^〉,

Because both types of linkers generate roughly the same maximal force for the tug-of-war, the initial stretching of the motor linkers is hardly influenced by the absence of compression forces. Furthermore, the steady state probabilities *P* ^st^(*n*_+_, *n*_−_) for the different activity states are quite similar for both types of linkers, see Fig. 8d, which implies that the overall cargo transport depends only weakly on the precise form of elastic coupling. For both types of linkers, the activity state (*n*_+_, *n*_−_) = (2, 0) with two actively pulling kinesins has the highest steady state probability.

Because the elastically coupled motors generate a higher steady state force for harmonic linkers compared to cable-like linkers, the average mechanical work 〈*W*_out_〉 performed by the harmonically coupled motors should also be larger. Thus, it is tempting to speculate that cable-like linkers lead to a larger dissipation of chemical free energy, as well. However, for an accurate analysis of this free energy transduction, one needs to calculate the overall mechanical work done by the whole system, including contributions from all activity states, which is beyond the scope of the present study.

## Discussion

We introduced a general theory for two antagonistic teams of motors that are coupled elastically to a common cargo or bead. Our theory reveals the complex and nested structure of the motors’ state space, consisting of multiple activity states and a large number of elastic substates, even for the relatively small system of two identical motors working against one antagonistic motor, see Figs 2 and 3. We then focussed on this latter system because it provides the simplest case with force sharing between identical motors. Our results for force sharing and force generation between kinesin and dynein motors are summarized in Figs 4 and 5. In all cases, we find that the most likely motor configuration in the steady state is characterized by equal force sharing. In addition, this form of equal force sharing applies even to the whole time-dependent evolution of the probability distributions towards their steady states as illustrated in Fig. 7. The same behavior is found when the motor linkers are described by nonharmonic cable-like springs, see Figs 8a and b. We find that the timescales for force generation and force relaxation during a tug-of-war are surprisingly similar for the harmonic and nonharmonic linkers, whereas the steady state forces are slightly lower for the latter case. The overall cargo transport, on the other hand, remains largely unaffected by the different types of linkers, see Fig. 8d. Furthermore, mutual unbinding of the elastically coupled motors from the filament leads to a reduction of the average forces compared to the force values predicted by the non-elastic model developed in previous studies^5,39^. The difference in the predictions of the elastic and non-elastic models becomes quite dramatic for motors with a high stall force value as illustrated in Fig. 5c. On the other hand, if teams of weak motors are involved in a tug-of-war, both models predict similar force values as in Fig. 5b. In the limit of small unbinding rates, the average elastic forces coincide with the force values predicted by the non-elastic model, see Fig. 6.

Another striking consequence of strain-induced unbinding is the non-monotonic build-up of the average elastic forces as displayed in Figs 7 and 8c. Inspection of these figures reveals that the average elastic forces acting on the motors exhibit maxima at intermediate times. These maxima disappear in the limit of small unbinding rates as shown in the SI, thereby directly demonstrating the intimate relation between the non-monotonic build-up of the elastic strain and the mutual strain-induced unbinding of the motors. For the case of nonharmonic cable-like motor linkers, the increased number of available rebinding states for the detached motors, see Fig. 8a4, might indicate that the mechanical work done by the motors is reduced compared to harmonically coupled motors. However, the overall free energy transduction requires a careful analysis of the overall mechanical work produced by the system and depends both on the hydrolysis rate and on the reaction free enthalpy of hydrolysis.

Our computational approach can be extended to elucidate other motor systems. One example is provided by motor teams that work against the force generated by an optical trap. In the latter context, sub-additive force generation by several motors has been experimentally observed using teams of up to four kinesin motors^20,21^. When applied to teams of kinesin motors, our theory also leads to sub-additive force generation whereas teams of other types of motors can generate force in an additive or even super-additive manner, as will be described elsewhere.

Another aspect that requires further study is the force-dependence of dynein’s unbinding rate. In the present study, we assumed a simple exponential force-dependence corresponding to slip bonds. However, both catch-bond behavior^8,18,23^ and slip-bond behavior^40^ have been proposed. Furthermore, it has also been observed that when dynein forms a complex with dynactin and the cargo adaptor Bicaudal-D2^29,41,42^, or in the presence of dynein-interacting protein LIS1 in a motor-cargo complex^43^, the processivity and force output of these complexes are enhanced. These additional properties can be implemented in our framework by adjusting the force-dependent parameters of the single motors.

Our model can also be used to study cooperative transport by two teams of motors that have the same polarity but move with different velocities^44–46^ or, more generally, by mixed teams that consist of motors with different single motor properties. The theoretical predictions presented here can be scrutinized experimentally by controlling the number and types of motors using preparation methods based, *e.g*., on DNA scaffolds and quantum dots as developed recently^20,21,30,47,48^.

## Model and Methods

A detailed description of the model and the computational methods are provided in the sections *S1 Theoretical description of elastically coupled motors, S2 Complete state space for cargo transport by (2* + *1) motors*, and *S4 Non-elastic model for (2* + *1) motors* in the SI. Single motor properties and the parameter values used in this study are presented below.

### Single motor properties

We describe the directed motion of a single motor bound to the filament as a one-dimensional random walk with forward and backward stepping rates *α* and *β*. The stepping rates determine the average motor velocity *v via* $\alpha -\beta =v/\ell$, where $\ell$ is the step size of the motor. In the absence of an external force *F*, the motor has the velocity *v*(*F* = 0) = *v*_0_. A resisting force *F* > 0 decreases the motor’s velocity *v*_0_ until the motor stalls at the stall force *F*_*s*_ with *v*(*F*_*s*_) = 0. For *F* > *F*_*s*_ the motor steps backwards and reaches the backward velocity −*v*_min_ for large positive *F*-values. For assisting forces *F* < 0, on the other hand, the velocity reaches its maximal value defined by *v*_max_ for large negative *F*-values. The force-velocity relationship *v*(*F*) can be parametrized in terms of the velocity parameters *v*_0_, *v*_min_ and *v*_max_ as well as the stall force *F*_*s*_^27,28^ for each motor species, which has the explicit form

7 $$v(F)=\frac{{v}_{{\rm{\max }}}\frac{{v}_{{\rm{\min }}}-{v}_{0}}{{v}_{0}-{v}_{{\rm{\max }}}}+{v}_{{\rm{\min }}}{(\frac{{v}_{{\rm{\max }}}}{{v}_{{\rm{\min }}}}\frac{{v}_{0}-{v}_{{\rm{\min }}}}{{v}_{0}-{v}_{{\rm{\max }}}})}^{F/{F}_{s}}}{\frac{{v}_{{\rm{\min }}}-{v}_{0}}{{v}_{0}-{v}_{{\rm{\max }}}}+{(\frac{{v}_{{\rm{\max }}}}{{v}_{{\rm{\min }}}}\frac{{v}_{0}-{v}_{{\rm{\min }}}}{{v}_{0}-{v}_{{\rm{\max }}}})}^{F/{F}_{s}}}.$$

We use the following sign convention to describe the force-velocity relations of both types of motors: *v*^±^(*F*) = ±*v*(*F*), where the plus or minus superscript indicates a plus or minus motor. In order o obtain an expression for the stepping rates of a single motor, we first define the force-dependent forward-to-backward stepping ratio *q*(*F*) of a single motor. This ratio depends exponentially on the external force and was fitted by^27,28,49^

8 $$q(F)={q}_{0}^{1-F/{F}_{s}},$$

with the zero-force stepping ratio *q*_0_ = 800 for kinesin. For dynein, we use the estimate *q*_0_ = 4^28^, in agreement with the stepping probabilities of yeast dynein measured by the Vale lab^31,32^. Note that at the stall force *F* = *F*_*s*_ the stepping ratio becomes *q*(*F*_*s*_) = 1, implying equal probabilities for the forward and backward steps of the motor. The force-dependent stepping rates of the motor are then defined by^27,28^

9 $$\begin{array}{ccc}\alpha (F)\equiv \frac{v(F)}{\ell }\,\frac{q(F)}{q(F)-1}\,, & {\rm{and}} & \beta (F)\equiv \frac{v(F)}{\ell }\frac{1}{q(F)-1},\end{array}$$

for forward and backward steps, respectively, where $\ell$ is the step size of the motor. Observe that equation (9) implies $\alpha (F)-\beta (F)=v(F)/\ell$.

In the absence of an external force, a bound motor detaches from the filament because of thermal fluctuations with a constant zero-force detachment rate *ε*_0_. External forces increase this unbinding rate exponentially as

10 $$\varepsilon (F)={\varepsilon }_{0}\exp (|F|/{F}_{d}),$$

where *F*_*d*_ is the detachment force determined by the energy barrier between the bound and the unbound state of the motor. A detached motor can rebind to the filament with the binding rate *π*_0_ which is taken to be independent of external forces acting on the cargo, *i.e*., *π*(*F*) = *π*_0_, because the motor remains in a relaxed state when it is detached from the filament^4^. See Table 1 for the parameter values used in this study.

## Supplementary information

Force sharing and force generation by two teams of elastically coupled molecular motors - Supplementary Information

## Data Availability

All data generated or analysed during this study are included in this published article and its Supplementary Information files.
