# Supplementary material for: Force sharing and force generation by two teams of elastically coupled molecular motors
Source: Sci Rep. 2019 Jan 24;9:454. doi: 10.1038/s41598-018-37126-0 (PMC6345805; doi:10.1038/s41598-018-37126-0)
Supplement: Supplementary file 1 — Force sharing and force generation by two teams of elastically coupled molecular motors - Supplementary Information [file 41598_2018_37126_MOESM1_ESM.pdf]

Force sharing and force generation by two teams of elastically coupled molecular motors - Supplementary Information

Mehmet Can Uçar and Reinhard Lipowsky
Max Planck Institute of Colloids and Interfaces, Science Park Golm, 14424 Potsdam, Germany
December 10, 2018

S1 Theoretical description of elastically coupled motors

Here we present our general theoretical framework that describes elastic interactions between an arbitrary number of motors, taking into account all possible mechanical steps performed by any motor in the system. We first define a one-dimensional Cartesian coordinate x parallel to the filament. Large positive values of x denote the plus end of the filament, and large negative values the minus end. We consider the activity state (n_-, n_+) of the system corresponding to a cargo that is simultaneously pulled by n_- minus and n_+ plus motors.

We label the minus motors by $i = 1, 2, \ldots, n_-$, and the plus motors by $i = n_- + 1, n_- + 2, \ldots, n_- + n_+$. The position of the motor i at the filament is given by $x = x_i$. The motors are attached to the cargo by linkers that act as elastic springs. The separation between the i-th motor and the cargo is then defined by

$$y_i \equiv x_i - x_{\text{ca}} \,. \tag{S1}$$

Note that the separation y_i is negative if the motor i is located between the cargo and the minus end of the filament and positive otherwise. The elastic linker of the i-th motor has the rest length $L_\parallel$ and the spring constant κ_i. To describe the elastic interaction forces between the motors as transmitted by the motor-cargo linkers, we will explicitly consider two types of force potentials corresponding (i) to harmonic springs, and (ii) to nonharmonic cable-like springs.

S1.1 Motors and cargo elastically coupled by harmonic springs

S1.1.1 General aspects of harmonic spring coupling

For harmonic springs, the elastic force $F_{i,\text{ca}}$ that motor i exerts onto the cargo is taken to be

$$F_{i,\text{ca}} = \kappa_i \left[y_i - \text{sgn}(y_i) L_\parallel \right] \,, \tag{S2}$$

with the sign function $\text{sgn}(y_i)$ that takes the values $\text{sgn}(y_i) = +1$ for $y_i > 0$ and $\text{sgn}(y_i) = -1$ for $y_i < 0$. This piece-wise linear force corresponds to a spring potential $V(y_i)$ of the form

$$V(y_i) = \tfrac{1}{2}\kappa_i (y_i - L_\parallel)^2 \quad \text{for} \quad y_i > 0 \,, \tag{S3}$$

and

$$V(y_i) = \tfrac{1}{2}\kappa_i(y_i + L_\|)^2 \quad \text{for} \quad y_i < 0\,. \tag{S4}$$

This piecewise harmonic potential has two minima located at $y_i = +L_\|$ and $y_i = -L_\|$, which are separated by a "sharp" barrier. Such a piece-wise harmonic potential may be obtained from a smooth symmetric double-well potential when we consider the limiting case in which the potential barrier between the two potential wells develops a kink.

The elastic force given by Eq. (S2) has a discontinuity at $y_i = 0$ corresponding to $x_i = x_{\rm ca}$. Indeed, one has

$$F_{i,\rm ca}(y_i = 0 - \epsilon) \approx +\kappa_i L_\| \quad \text{and} \quad F_{i,\rm ca}(y_i = 0 + \epsilon) \approx -\kappa_i L_\| \tag{S5}$$

for small ϵ. This discontinuous behavior applies to a real spring between the motor and the cargo when we assume that the cargo has a fixed perpendicular distance from the filament and when we focus on the force component parallel to the filament. This spring is compressed for small negative y_i-values with $-L_\| < y_i < 0$ and then pushes the cargo to the right. For small positive y_i-values with $0 < y_i < +L_\|$, the spring is also compressed but now pushes the cargo to the left. Therefore, such a spring exerts a positive force for small negative y_i-values and a negative force for small positive y_i-values which implies that the spring is unstable and "snaps" at $y_i = 0$.

Now, consider a plus motor (kinesin) with label $n_- + 1 \le i \le n_- + n_+$. If this motor is located at $x_i > x_{\rm ca}$, it would have to perform several backward steps, for fixed cargo position $x_{\rm ca}$, in order to attain a position with $x_i < x_{\rm ca}$. More precisely, if the plus motor started from a position close to the relaxed position with $y_i = L_\|$, it would have to perform about $L_\|/\ell$ backward steps in order to get to the other side of the cargo. This is not impossible but rather unlikely, at least for $L_\|/\ell \gg 1$. Because the motor stalk is about 80 nm in length and the step size is 8 nm, the length ratio $L_\|/\ell$ is of the order of 10 and we can then ignore motor states of the plus motors with $x_i < x_{\rm ca}$ or $y_i < 0$. For the same reason, we can also ignore motor states of the minus motors with $x_i > x_{\rm ca}$ or $y_i > 0$.

In principle, we could also consider initial motor configurations for which a minus motor is located to the right of the cargo (or a plus motor to the left of the cargo). The minus motor would then experience an elastic force that pushes it, for fixed cargo position, towards $y_i = +L_\|$. Depending on the height of the barrier of the piecewise harmonic potential, the motor could then be kinetically trapped in such a state. The latter situation would arise if the elastic force $\kappa L_\|$ that pushes the minus motor in the backward direction (towards the plus end) for small positive y_i-values is large compared to the stall force F_s of the minus motor.

In the following, we will focus on initial motor configurations within the [−ca+] sectors for which the minus motors are located to the left of the cargo, *i.e.*, between the cargo and the minus end of the filament while the plus motors are located to the right of the cargo, *i.e.*,

$$x_i < x_{\rm ca} \quad \text{for} \quad i = 1, \ldots, n_-\,, \tag{S6}$$

and

$$x_{\rm ca} < x_i \quad \text{for} \quad i = n_- + 1, \ldots, n_- + n_+ \,, \tag{S7}$$

In this way, we will avoid any "snapping" of the elastic linkers as well as any kinetic trapping of the motors on the side of the cargo opposite to their polarity. As explained in subsection S1.2 below, we can omit the constraints on the ordering of the motor positions as given by Eq. (S6) and Eq. (S7) if we consider nonharmonic, cable-like linkers.

S1.1.2 Force balance and cargo position

For motor configurations within the [−ca+] sector, the elastic force $F_{{\rm ca},i}$ that the cargo exerts onto the motor i is given by

$$F_{{\rm ca},i} = -F_{i,{\rm ca}} \,. \tag{S8}$$

It then follows from Eq. (S2) that

$$F_{{\rm ca},i} = -\kappa_i (y_i + L_{||}) \quad \text{for the minus motors,} \tag{S9}$$

and

$$F_{{\rm ca},i} = -\kappa_i (y_i - L_{||}) \quad \text{for the plus motors.} \tag{S10}$$

We now use the convention that resisting forces acting against the preferred direction of a motor are taken to be positive, and assisting forces are defined to be negative. In addition, we define the elastic displacements

$$u_i \equiv -(y_i + L_{||}) \quad \text{for the minus motors,} \tag{S11}$$

and

$$u_i \equiv y_i - L_{||} \quad \text{for the plus motors.} \tag{S12}$$

As a consequence, the elastic force F_i experienced by the motor i assumes the simple form

$$F_i = \kappa_i u_i \quad \text{for both plus and minus motors.} \tag{S13}$$

The sign convention implies that $F_i = F_{{\rm ca},i}$ for minus motors and $F_i = -F_{{\rm ca},i}$ for plus motors.

We now denote the spring constants of the minus and plus motors by κ_- and κ_+, *i.e.*,

$$\kappa_i \equiv \kappa_- \quad \text{for} \quad i = 1, \ldots, n_-, \tag{S14}$$

and

$$\kappa_i \equiv \kappa_+ \quad \text{for} \quad i = n_- + 1, \ldots, n_- + n_+ \,. \tag{S15}$$

The force balance condition as required by Newton's third law then has the form

$$\sum_{i=1}^{n_-} \kappa_- u_i = \sum_{i=n_-+1}^{n_-+n_+} \kappa_+ u_i \tag{S16}$$

When all motors have the same spring constant $\kappa = \kappa_- = \kappa_+$, the force balance condition simplifies and becomes

$$\sum_{i=1}^{n_-} u_i = \sum_{i=n_-+1}^{n_-+n_+} u_i \,, \tag{S17}$$

that is, the sum over all elastic displacements of the minus motors is equal to the sum over all elastic displacements of the plus motors. The latter relation implies that the elastic state of the cargo-motor complex can be described by $n_- + n_+ - 1$ independent displacement variables u_i.

Substituting Eqs. (S1) and (S11-S13) into the force balance condition in Eq. (S16), we obtain the cargo position

$$x_{\rm ca} = \varphi \left(\kappa_- \sum_{i=1}^{n_-} x_i + \kappa_+ \sum_{i=n_-+1}^{n_-+n_+} x_i + L_{\|} \left(n_- \kappa_- - n_+ \kappa_+ \right) \right) \,, \tag{S18}$$

with the prefactor

$$\varphi \equiv \frac{1}{n_- \kappa_- + n_+ \kappa_+} \,. \tag{S19}$$

S1.1.3 Single motor steps and elastic displacements

When one of the motors with label $i = j$ performs a single mechanical step, the motor positions x_i change according to

$$x_j \to x'_j = x_j \pm \ell \equiv x_j + \Delta x_j \tag{S20}$$

and

$$x_i \to x'_i = x_i \quad \text{for} \quad i \neq j \,, \tag{S21}$$

i.e., the positions of the non-stepping motors remain unchanged. In Eq. (S20) the coordinate change $\Delta x_j = +\ell$ corresponds to a forward step of a plus motor or to a backward step of a minus motor, whereas $\Delta x_j = -\ell$ describes a backward step of a plus motor or a forward step of a minus motor.

Any motor step will affect the cargo position $x_{\rm ca}$. It follows from Eq. (S18) that the cargo position $x_{\rm ca}$ will be shifted according to

$$x_{\rm ca} \to x'_{\rm ca} = x_{\rm ca} + \Delta x_{\rm ca} \quad \text{with} \quad \Delta x_{\rm ca} \equiv \Delta x_j \, \varphi \, \kappa_+ \tag{S22}$$

if the stepping motor j is a plus motor, and according to

$$x_{\rm ca} \to x'_{\rm ca} = x_{\rm ca} + \Delta x_{\rm ca} \quad \text{with} \quad \Delta x_{\rm ca} \equiv \Delta x_j \, \varphi \, \kappa_- \tag{S23}$$

if the stepping motor j is a minus motor. Thus, a single step of a plus motor shifts the cargo position by $\pm \ell \, \varphi \, \kappa_+$ while a single step of a minus motor shifts this position by $\pm \ell \, \varphi \, \kappa_-$, with φ as given by Eq. (S19). The latter expression for φ implies that the cargo shift depends on the

motor numbers n_- and n_+ as well as on the elastic spring constants κ_- and κ_+ of the two motor teams.

The coordinate change Δx_j of the stepping motor together with the cargo shift $\Delta x_{\rm ca}$ modify the elastic displacements u_i of all motors. If the stepping motor j is a *plus* motor, its own elastic displacement is changed according to

$$u_j \to u'_j = u_j + \Delta x_j \,(1 - \varphi\,\kappa_+)\,, \tag{S24}$$

whereas the elastic displacements of the nonstepping motors behave as

$$u_i \to u'_i = u_i - \Delta x_j\,\varphi\,\kappa_+ \quad \text{for a plus motor with} \quad i \neq j\,, \tag{S25}$$

and

$$u_i \to u'_i = u_i + \Delta x_j\,\varphi\,\kappa_+ \quad \text{for all minus motors.} \tag{S26}$$

Likewise, if the stepping motor j is a *minus* motor, the elastic displacements are transformed according to

$$u_j \to u'_j = u_j + \Delta x_j \,(1 - \varphi\,\kappa_-) \quad \text{for the stepping motor } j\,, \tag{S27}$$

and according to

$$u_i \to u'_i = u_i - \Delta x_j\,\varphi\,\kappa_- \quad \text{for a minus motor with} \quad i \neq j\,, \tag{S28}$$

and

$$u_i \to u'_i = u_i + \Delta x_j\,\varphi\,\kappa_- \quad \text{for all plus motors.} \tag{S29}$$

S1.1.4 Elastic substates of the activity state (n_-, n_+)

We now consider the elastic substates of the activity state (n_-, n_+), corresponding to all possible configurations of n_- minus and n_+ plus motors that are all bound to the discrete binding sites of the filament. Each of these elastic substates is described by the elastic displacements u_i of all motors. These elastic displacements define the $(n_- + n_+)$-dimensional vector

$$\mathbf{u} \equiv (u_1, u_2, \ldots, u_{n_-+n_+}) \tag{S30}$$

and the *relaxed* reference state

$$\mathbf{u}_0 \equiv (u_1 = 0, u_2 = 0, \ldots, u_{n_-+n_+} = 0)\,. \tag{S31}$$

The vectors $\mathbf{u}$ form an $(n_- + n_+)$-dimensional vector space with its origin at $\mathbf{u} = \mathbf{u}_0$.

Note that, in general, the rest length $L_\|$ and the step size ℓ are two independent length scales. Therefore, the motor-cargo system will attain a minimal strain substate $\mathbf{u}_{\rm ms} \neq \mathbf{u}_0$ unless the rest length $L_\|$ and the step size ℓ are commensurate in the sense that $2L_\| = k^*\ell$ with some integer

k^*. We focus on such a commensurate situation in order to eliminate the rest length $L_\parallel$ as a parameter.

Furthermore, we define the $(n_- + n_+)$-dimensional unit vectors

$$\hat{\mathbf{e}}_i \equiv (0, 0, \ldots, u_i = 1, \ldots, 0)\,, \tag{S32}$$

which can be used to decompose the elastic substates into different components *via*

$$\mathbf{u} = \sum_i u_i \hat{\mathbf{e}}_i\,. \tag{S33}$$

Starting from an arbitrary substate $\mathbf{u}$, a single mechanical step of a *plus* motor with label j leads to the new elastic substate

$$\mathbf{u}' = \mathbf{u} \pm \ell\, \mathbf{b}_j \tag{S34}$$

with the vector

$$\mathbf{b}_j = \hat{\mathbf{e}}_j + \varphi\, \kappa_+ \left[\sum_{k=1}^{n_-} \hat{\mathbf{e}}_k - \sum_{k=n_-+1}^{n_-+n_+} \hat{\mathbf{e}}_k \right] \quad \text{for } j > n_-\,, \tag{S35}$$

where the plus and minus sign in Eq. (S34) corresponds to a forward and a backward step of the plus motor j, respectively.

Similarly, a single mechanical step of a *minus* motor with label j leads to the new substate

$$\mathbf{u}' = \mathbf{u} \pm \ell\, \mathbf{b}_j \tag{S36}$$

with the vector

$$\mathbf{b}_j = \hat{\mathbf{e}}_j + \varphi\, \kappa_- \left[-\sum_{k=1}^{n_-} \hat{\mathbf{e}}_k + \sum_{k=n_-+1}^{n_-+n_+} \hat{\mathbf{e}}_k \right] \quad \text{for } j \leq n_-\,, \tag{S37}$$

where the plus and minus sign in Eq. (S36) correspond to a forward and a backward step of the minus motor j, respectively.

Using the vectors $\mathbf{b}_j$ defined by Eqs. (S35) and (S37), we can now describe all possible elastic substates of the activity state (n_-, n_+). Starting from the relaxed state $\mathbf{u}_0$ in Eq. (S31), these substates have the form

$$\mathbf{u} = \mathbf{u}_0 + \ell \sum_{j=1}^{n_-+n_+} s_j\, \mathbf{b}_j = \ell \sum_{j=1}^{n_-+n_+} s_j\, \mathbf{b}_j\,, \tag{S38}$$

with integer s_j, and represent a lattice in $\mathbf{u}$-space. Positive values of s_j correspond to s_j successive forward steps of motor j, negative values of s_j to $|s_j|$ successive backward steps of motor j. Therefore, we can express each displacement vector $\mathbf{u}$ as a superposition of the $(n_- + n_+)$ vectors $\mathbf{b}_j$.

Furthermore, because of the force balance condition in Eq. (S16), the elastic displacements u_i satisfy one linear equation and are, thus, not linearly independent. Likewise, the $(n_- + n_+)$ vectors $\mathbf{b}_j$ satisfy the linear relation

$$\sum_{j=1}^{n_-} \mathbf{b}_j = \sum_{j=n_-+1}^{n_-+n_+} \mathbf{b}_j \tag{S39}$$

which implies

$$\mathbf{b}_{n_-+n_+} = \sum_{j=1}^{n_-} \mathbf{b}_j - \sum_{j=n_-+1}^{n_-+n_+-1} \mathbf{b}_j \,. \tag{S40}$$

Therefore, all displacement vectors $\mathbf{u}$ can be expressed as linear combinations of the reduced set of vectors $\mathbf{b}_j$ with $j \neq n_- + n_+$. This reduced set spans a $(n_- + n_+ - 1)$-dimensional hyperplane within the $(n_- + n_+)$-dimensional vector space with basis vectors $\hat{\mathbf{e}}_i$.

S1.1.5 Elastic substates for two minus motors and one plus motor

For the activity state $(n_-, n_+) = (2, 1)$ with two minus motors and one plus motor bound to the filament, the vectors $\mathbf{b}_j$ defined in Eqs. (S35) and (S37) have the form

$$\mathbf{b}_1 = \begin{pmatrix} 1 - \varphi\,\kappa_- \\ -\varphi\,\kappa_- \\ \varphi\,\kappa_- \end{pmatrix}, \; \mathbf{b}_2 = \begin{pmatrix} -\varphi\,\kappa_- \\ 1 - \varphi\,\kappa_- \\ \varphi\,\kappa_- \end{pmatrix}, \quad \text{and} \quad \mathbf{b}_3 = \begin{pmatrix} \varphi\,\kappa_+ \\ \varphi\,\kappa_+ \\ 1 - \varphi\,\kappa_+ \end{pmatrix} \tag{S41}$$

with $\varphi = 1/(n_-\kappa_- + n_+\kappa_+)$ as in Eq. (S19) and $\mathbf{b}_1 + \mathbf{b}_2 = \mathbf{b}_3$. Therefore, the displacement vectors $\mathbf{u}$ are located on a 2-dimensional plane within the 3-dimensional Euclidean space. For $\kappa_- = \kappa_+ \equiv \kappa$, we obtain $\varphi\,\kappa_- = \varphi\,\kappa_+ = 1/3$ and the vectors $\mathbf{b}_j$ in Eq. (S41) become identical with Eqs. (2) and (3) in the main text.

S1.2 Motors and cargo elastically coupled by cable-like springs

We now model the motor linkers as cable-like springs, which behave like harmonic springs when stretched, but do not generate compressional forces. As before, the minus motors are labeled by $i = 1, 2, \ldots, n_-$, the plus motors by $i = n_- + 1, n_- + 2, \ldots, n_- + n_+$. Motor i is again located at $x = x_i$ and the cargo at $x = x_{\text{ca}}$. The separation between motor i and the cargo is still given by $y_i = x_i - x_{\text{ca}}$ as in Eq. (S1). The force $F_{\text{ca},i}$ that the cargo exerts onto motor i is now given by

$$\begin{aligned} F_{\text{ca},i} &= -\kappa_i(y_i + L_\|) && \text{for } y_i < -L_\|, \\ &= 0 && \text{for } -L_\| < y_i < +L_\|, \text{ and} \\ &= -\kappa_i(y_i - L_\|) && \text{for } y_i > +L_\|. \end{aligned} \tag{S42}$$

with spring constant κ_i of the cable-like linker between the cargo and motor i. The associated force potential has the form

$$\begin{aligned} V(y_i) &= \tfrac{1}{2}\,\kappa_i(y_i + L_\|)^2 \quad \text{for } y_i < -L_\|, \\ &= 0 \quad\quad\quad\quad\quad\;\; \text{for } -L_\| < y_i < +L_\|, \text{ and} \\ &= \tfrac{1}{2}\,\kappa_i(y_i - L_\|)^2 \quad \text{for } y_i > +L_\|. \end{aligned} \tag{S43}$$

The latter potential can be obtained from the piece-wise harmonic potential defined by Eqs. (S3) and (S4) by replacing the compressional part for $-L_\| < y_i < +L_\|$ by $V(y_i) = 0$. As a result of this replacement, we obtain the elastic force $F_{\text{ca},i}$ as given by Eq. (S42), which is now continuous for all values of y_i. Using the Heaviside step function $\Theta(x)$ with $\Theta(x) = 0$ for $x < 0$ and $\Theta(x) = 1$ for $x \leq 0$, the elastic force in Eq. (S42) can be rewritten in the more concise form

$$F_{\text{ca},i} = -\kappa_i \left[(y_i - L_\|)\Theta(y_i - L_\|) + (y_i + L_\|)\Theta(-y_i - L_\|) \right] . \tag{S44}$$

When a *minus* motor with $i \leq n_-$ moves in the force potential $V(y_i)$ as given by Eq. (S43), it will typically be located in the vicinity of $y_i = -L_\|$. Indeed, when the motor is closer to the cargo, it does not experience any force and thus makes forward steps corresponding to its zero-force velocity until it reaches $y_i = -L_\|$. Therefore, we will again define the elastic displacement u_i of a minus motor by $u_i = -y_i - L_\|$ as in Eq. (S11). Together with our previously mentioned convention that a resisting force experienced by a motor is positive, we then obtain the single-motor force

$$F_i = F_{\text{ca},i} = \kappa_i \left[u_i\Theta(u_i) + (2L_\| + u_i)\Theta(-2L_\| - u_i) \right] \quad \text{for} \quad i = 1, 2, \ldots, n_- . \tag{S45}$$

Likewise, when a *plus* motor with $i > n_-$ moves in the same force potential, it will typically be located in the vicinity of $y_i = +L_\|$. Using the elastic displacement $u_i = y_i - L_\|$ as in Eq. (S12) together with our convention about the positive sign for resisting forces, the single-motor force F_i acting on a plus motor has the form

$$F_i = -F_{\text{ca},i} = \kappa_i \left[u_i\Theta(u_i) + (2L_\| + u_i)\Theta(-2L_\| - u_i) \right] \quad \text{for} \quad i = n_- + 1, \ldots, n_- + n_+ . \tag{S46}$$

Thus, when expressed in terms of the elastic displacements u_i, the single-motor force acting on a plus motor has again the same form as for a minus motor.

The force balance condition has the same form as Eq. (S16), *i.e.*,

$$\sum_{i=1}^{n_-} F_i = \sum_{i=n_-+1}^{n_-+n_+} F_i \, ,$$

but with the forces now given by Eqs. (S45) and (S46). If all $(n_- + n_+)$ motors have non-relaxed linkers, *i.e.*, for $u_i > 0$ or $u_i < -2L_\|$ for all i, one obtains the cargo position x_{ca} as given by Eq. (S18). In general, however, we need to examine for each motor i whether it has a relaxed linker or not, which leads to a total number of $2^{n_- + n_+}$ cases for the force balance condition.

One can then determine the cargo position x_{ca} and the prefactor φ, see Eq. (S19), for these different cases and calculate the elastic displacements u_i for each mechanical step. In contrast to harmonic springs, the plus and minus motors can now attain any position relative to the cargo and the ordering of these motors is no longer constrained by Eqs. (S6) and (S7).

S2 Complete state space for cargo transport by (2+1) motors

The dynamics of the (2+1)-motor system is described as a continuous-time Markov process or master equation on a discrete state space. Such a process has the following basic ingredients: the activity states of the motor system, the elastic substates, and the possible transitions between the states and substates. Furthermore, the different transitions are characterized kinetically by certain transition rates which depend, in general, on forces experienced by the motors.

As mentioned in the main text, the system with $N_- = 2$ minus and $N_+ = 1$ plus motors can dwell in 8 different activity states with $n_- \leq 2$ minus and $n_+ \leq 1$ plus motors simultaneously pulling the cargo, see Fig. 2 of the main text. When all motors are detached from the filament, *i.e.*, for $n_- = n_+ = 0$, the motor-cargo complex remains in a diffusive state until one of the available motors rebinds to the filament. In activity states with a single motor pulling on the cargo, *i.e.*, for $(n_-, n_+) = (1,0)_1, (1,0)_2$ or $(0,1)$, Eqs. (S1) and (S11-S13) with Eq. (S18) imply that the elastic force acting on the single motor vanishes. Thus, only activity states with $n = n_- + n_+ \geq 2$ include elastic substates that correspond to single mechanical steps of the bound motors, compare Fig. 3 in the main text.

The three-motor activity state $(n_-, n_+) = (2,1)$ consists of elastic substates $\{r_1, r_2\}$, where $r_1 = s_1 + s_3$ and $r_2 = s_1 + s_3$ are the displacement numbers, corresponding to different motor configurations. These configurations are connected by transitions arising from single steps of individual motors, see left box of Fig. 3 in the main text. In the latter box, horizontal and vertical edges correspond to forward and backward steps of one of the two minus motors, and the diagonal edges to stepping events of the plus motor. The stepping transitions are governed by the forward and backward stepping rates $\alpha(F)$ and $\beta(F)$, see Eq. (9) in the main text. Note that the forces acting on the three motors in the elastic substate $\{r_1, r_2\}$ are given by $\mathbf{F}\{r_1, r_2\} = (F_1, F_2, F_3) = \kappa\,\mathbf{u}\{r_1, r_2\}$. Therefore, the stepping rates depend on the elastic substate and can be different for each motor in that substate.

S2.1 Force dependent transition rates

During a single mechanical step, the elastic force acting on the motor changes monotonically from its initial value before the step to its final value after the step [1], hence the effective force acting on the motor is given by the arithmetic mean of these values. In the activity state (2,1), for instance, the effective force acting on the minus motor $j = 1$ during a forward step is given

by

$$\begin{aligned}\bar{F}_>(u_1) &= \tfrac{1}{2}\kappa\ell\left(\frac{2r_1 - r_2}{3} + \frac{2(r_1+1) - r_2}{3}\right) \\ &= F(u_1) + F_\kappa/3\,,\end{aligned} \tag{S47}$$

where $F_\kappa \equiv \kappa\ell$ is the elastic strain force corresponding to an extension of a motor linker by ℓ. Similar calculations for backward steps and for all remaining motors define the effective forces acting on the j-th motor by

$$\bar{F}_>(u_j) \equiv\; F(u_j) + F_\kappa/3\,,\ \text{during a forward step, and} \tag{S48}$$

$$\bar{F}_<(u_j) \equiv\; F(u_j) - F_\kappa/3\,,\ \text{during a backward step.} \tag{S49}$$

All forward and backward stepping rates in the activity state $(2,1)$ are then determined by $\alpha(\bar{F}_>(u_j))$ and $\beta(\bar{F}_<(u_j))$, respectively.

Any of the three bound motors can unbind from an elastic substate $\{r_1, r_2\}$ of the activity state $(2,1)$ with the unbinding rate

$$\epsilon^\pm(F_j) = \epsilon_0^\pm \exp(|F_j|/F_d^\pm)\,, \tag{S50}$$

where $F_d^\pm$ denotes the detachment force of a single plus or minus motor, and we used $F(u_j) \equiv F_j$ for notational clarity. Starting from the activity state (2,1), the detachment of a minus motor leads to the activity state $(n_-, n_+) = (1,1)$, describing a tug-of-war between a single minus and a single plus motor. As mentioned before, we distinguish the states $(1,1)_1$ and $(1,1)_2$ as well as the states $(1,0)_1$ and $(1,0)_2$ where the subscripts correspond to the minus motor labels $j = 1, 2$.

Evaluating the displacement vector $\mathbf{u}$ as defined by Eq. (S38) for the motors $i = 1$ and $j = 3$ in the activity state $(1,1)_1$, we obtain

$$\mathbf{u} = (u_1, u_3) = \ell\, r_1 \left(\tfrac{1}{2}\,, \tfrac{1}{2}\right)\,, \tag{S51}$$

such that the displacement number r_1 determines all possible elastic forces in this activity state. The same analysis applies for the tug-of-war between the motors $i = 2$ and $j = 3$, where the displacement number r_2 determines the elastic substates of the activity state $(1,1)_2$.

Starting from the activity state $(2,1)$, a detachment of the single plus motor leads to the activity state $(n_-, n_+) = (2,0)$. The latter state includes two identical motors bound to the filament, thus, its strain space is also determined by a single variable representing the steps of the motors. In this case the displacement vector $\mathbf{u}$ is evaluated as

$$\mathbf{u} = (u_1, u_2) = \ell\, r_{12} \left(\tfrac{1}{2}\,, -\tfrac{1}{2}\right)\,, \tag{S52}$$

where we introduced the variable $r_{12} \equiv r_1 - r_2 = s_1 - s_2$ and denote the elastic substates of the activity state $(2,0)$ by $\{r_{12}\}$.

As in Eqs. (S48) and (S49), the effective forces acting on the motors during stepping transitions between different elastic substates of the activity states $(1, 1)$ and $(2, 0)$ are given by

$$\bar{F}_{>}(u_j) \equiv F(u_j) + F_\kappa/2\,, \text{ during a forward step, and} \tag{S53}$$

$$\bar{F}_{<}(u_j) \equiv F(u_j) - F_\kappa/2\,, \text{ during a backward step,} \tag{S54}$$

where $j = 1, 3$ and $j = 2, 3$ for the two activity states $(1, 1)_1$ and $(1, 1)_2$, respectively, and $j = 1, 2$ for the state $(2, 0)$.

Further unbinding events from the two-motor bound states $(1, 1)_1$, $(1, 1)_2$ or $(2, 0)$ can lead to three single motor states: two single minus motor states $(1, 0)_1$ and $(1, 0)_2$, as well as one single plus motor state $(0, 1)$. When the process dwells in one of the single motor states, we assume that the single bound motor carries the cargo particle in a load-free manner. The rate at which it unbinds from the filament is thus determined by the zero-force unbinding rate $\epsilon_0^\pm$. We also include the unbound cargo state $(0, 0)$ in the state space, where all motors are detached from the filament.

Rebinding of a detached motor to the filament occurs with the force-independent binding rate $\pi_0^\pm$. Generally, when an unbound motor rebinds to the filament its linker will be in a relaxed state. This is the case for rebinding events from single motor states $(1, 0)_1$, $(1, 0)_2$, and $(0, 1)$ to two-motor states $(2, 0)$, $(1, 1)_1$ and $(1, 1)_2$. However, when a detached motor rebinds from one of the latter activity states with two bound motors, the elastic substate of the system prior to rebinding will have an effect on elastic substate at which the system arrives after rebinding.

Suppose, for example, that the system dwells in the elastic substate $\{r_1 = 3\}$ of the activity state $(1, 1)_1$, with the second minus motor being detached from the filament. It follows from Eq. (S51) that the elastic force acting on both bound motors is given by $F_1 = F_3 = 3\kappa\ell/2$. If the detached minus motor now rebinds to the filament, we would like to obtain a motor configuration for which the rebound motor is relaxed and the elastic forces acting on the previously bound motors remain unchanged. However, none of the elastic substates $\{r_1, r_2\}$ of the activity state $(2, 1)$ generates the elastic force vector $\mathbf{F} = (\frac{3}{2}\kappa\ell, 0, \frac{3}{2}\kappa\ell)$. The same situation arises for rebinding events from elastic substates of the activity states $(2, 0)$, $(1, 1)_1$ and $(1, 1)_2$, whenever the separation of the bound motors before the rebinding of the third motor is given by $(2k + 1)\ell$ with integer k. In such cases, we allow the detached motor to rebind to the "nearest" possible state such that its linker is either stretched or compressed by an amount of $\ell/3$, depending on the elastic substate prior to its rebinding. This rule generates a strain energy of $\kappa(\ell/3)^2 \simeq 1.4\,\text{pN nm}$ per rebinding event, which is about $k_\text{B}T/3$ at $T = 300\,\text{K}$.

S2.2 Markov process on complete state space for (2+1) motors

Starting from the complete state space corresponding to the network in Figs. 2 and 3 of the main text, we now define a Markov process on this network by a transition rate matrix $\mathbf{W}$ with the matrix elements $W_{ij} = \omega_{ij}$ for $i \neq j$, and to $W_{ii} = -\sum_k \omega_{ik}$. For a given choice of motor

parameters, see Table 1 in the main text, all transition rates ω_{ij} of the complete network are fixed, and the time evolution of the probability $p_i(t)$ to find the system in state i at time t is described by the master equation

$$\frac{\partial}{\partial t} p_i(t) = \sum_j \left[\omega_{ji} p_j(t) - \omega_{ij} p_i(t)\right] . \tag{S55}$$

The steady state probabilities as displayed in Fig. 4 in the main text satisfy $\frac{\partial}{\partial t} p_i(t) = 0$ and can be obtained by calculating the eigenvector of the associated transition rate matrix with eigenvalue zero. To define the transition rate matrix $\mathbf{W}$, we first make an ordered list of all states in the complete network as described in the previous section. For each state labelled by i we then consider all outgoing rates, which determine the diagonal elements $W_{ii} = -\sum_k \omega_{ik}$, and the off-diagonal elements $W_{ij} = \omega_{ij}$ for $i \neq j$. For the substates of the activity state (2,1), for instance, all outgoing rates are determined by the force-dependent forward and backward stepping rates $\alpha^{\pm}(F)$ and $\beta^{\pm}(F)$, as well as the unbinding rates $\epsilon^{\pm}(F)$ of each motor in the system, where the single-motor forces F are determined by the displacement vector $\mathbf{u}$, see Eq. (S38). Having defined all entries W_{ij} of the transition rate matrix, we then calculate its eigenvector corresponding to the eigenvalue zero using built-in functions in *Mathematica 9.0* [2].

To obtain the time-evolution of these probabilities as presented in Fig. 7 of the main text, we integrated the master Eq. (S55), forward in time using the asymptotic equality

$$p_i(t + \Delta t) \approx p_i(t) + \Delta t \, \mathbf{W} p_i(t) \tag{S56}$$

for small time intervals Δt. The latter expressions are evaluated numerically starting from the initial distribution $p_i(t = 0) = \delta_{ij_0}$ corresponding to the relaxed elastic substate $j_0 = \{s_1 = 0, s_2 = 0, s_3 = 0\}$ of the activity state $(2, 1)$. For the calculations in Fig. 7 and 8 of the main text, we used the time interval $\Delta t = 10^{-4}\,$s, and for the calculation in Fig. S1 the value $\Delta t = 10^{-3}\,$s.

S3 Motor teams with different spring constants

In the main text, we present our results for two opposing teams of motors with identical spring constants $\kappa_- = \kappa_+ = \kappa$. In general, however, the kinesin and dynein motors have a very different molecular structure and one might expect that the spring constants κ_- and κ_+ have different values. Here we investigate the effects of unequal spring constants for a tug-of-war between two dyneins against a single kinesin. We choose the values $\kappa_- = \kappa_+/4 = 0.05\,\text{pN/nm}$ for the dynein motors, in agreement with estimates from experimental data, see Refs. [3, 4].

For the case $\kappa_- \neq \kappa_+$, the prefactor φ, which determines the cargo shift at each mechanical step, depends on the $\kappa_\pm$-values, see Eq. (S19). In the activity state $(n_- = 2, n_+ = 1)$, for instance, it is given by $\varphi = 1/(2\kappa_- + \kappa_+) = 2/(3\kappa_+)$. A single step of the plus-directed kinesin now displaces the cargo by an amount $\varphi\,\kappa_+\,\ell = 2\ell/3$, as follows from Eq. (S22), whereas a

single step of one of the dyneins leads to a displacement of $\varphi\,\kappa_-\,\ell = \ell/6$. All force-dependent transitions in the complete state space are governed by the elastic forces $\mathbf{F}$, which determine the corresponding transition rates and can be evaluated using Eqs. (S13) and (S38) for each activity state (n_-, n_+), as described above for the case with $\kappa_- = \kappa_+$.

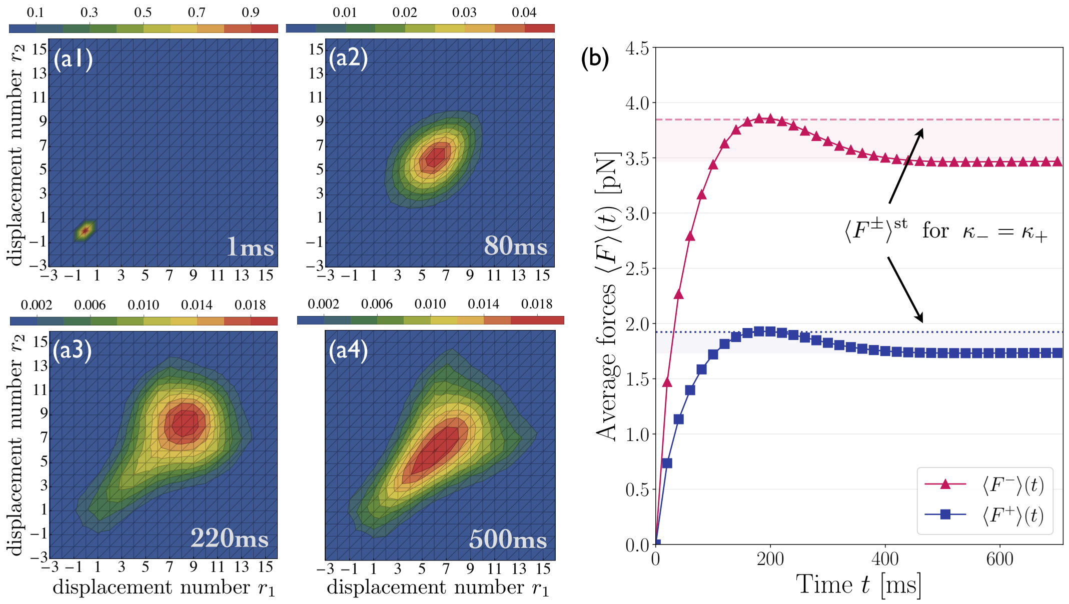

Figure S1: (a1-a4) Time evolution of the conditional probability distribution $\hat{p}\{r_1, r_2\}(t)$ for the elastic substates $\{r_1, r_2\}$ of the activity state (2,1) for two weak dyneins against one kinesin. The stiffness of dynein motors is set to be $\kappa_- = 0.05\,\text{pN/nm}$, which is 4-fold lower than kinesin's spring constant $\kappa_+ = 0.2\,\text{pN}$. The long-time limit of the probability distribution in (a4) displays tails towards high r_j-values, in contrast to the steady state distribution plotted in Fig. 4(b1) of the main text. (b) The time evolution of the average elastic forces $\langle F^\pm \rangle(t)$ generated during the tug-of-war exhibits a maximum at intermediate timescales, which is less pronounced than for $\kappa_- = \kappa_+$ see Figs. 7(b) and 8(c). The steady state values $\langle F^\pm \rangle^{\text{st}}$ slightly deviate from the corresponding values for $\kappa_- = \kappa_+$.

We now focus on the case of two weak dyneins and one kinesin with $\kappa_- = \kappa_+/4 = 0.05\,\text{pN/nm}$. In Figs. S1(a1-a4) we present the time evolution of the conditional probability distribution $\hat{p}\{r_1, r_2\}$ for the elastic substates $\{r_1, r_2\}$ of the activity state (2,1). The process converges to the steady state after approximately $t \simeq 400\,\text{ms}$, which exceeds the corresponding timescale of $t \simeq 300\,\text{ms}$ for the case of identical linkers (data not shown). We observe that the lower κ_- of the two dynein motors leads to a broadening of the probability distribution around the diagonal states, as illustrated in Fig. S1(a4). Interestingly, this broadening is different from the case of cable-like motor linkers, see Fig. 8(a4) of the main text. One might speculate that the low stiffness of the dynein motors with a large load-free velocity of $v_0 = 800\,\text{nm/s}$, see Table 1 in the main text, allows them to take a larger amount of successive forward steps as compared to the identical spring case. In contrast to the cable-like linker model, however, the number of

available rebinding sites are limited and thus the distribution does not spread towards negative r_j-values.

Furthermore, the time evolution of the average forces plotted in Fig. S1 displays a less pronounced maximum force and slightly lower asymptotic values than for identical spring constants. The latter feature is also observed for cable-like springs, see Fig. 8(c) in the main text. As in all other cases studied here, the most likely configuration is characterized by equal force sharing.

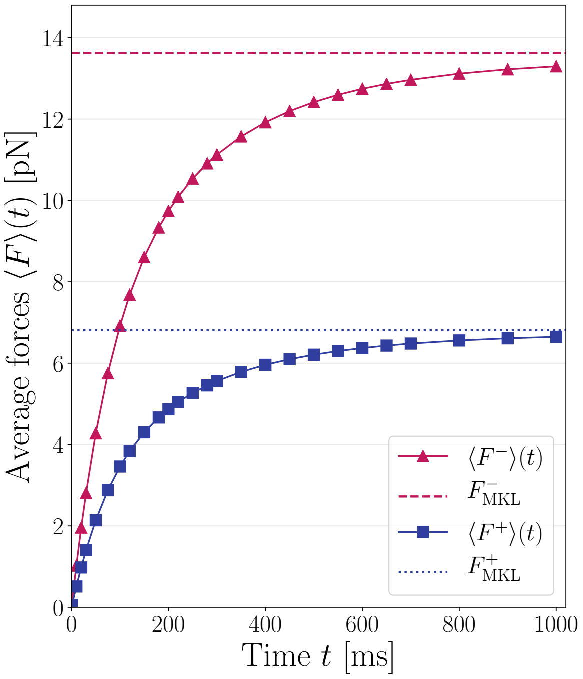

Figure S2: **Limit of small unbinding rates for the tug-of-war between two kinesins and one strong dynein:** Time evolution of the average elastic forces $\langle F^{\pm} \rangle(t)$ experienced by the motors for small unbinding rates as described by Eq. (10) of the main text with $\epsilon_0^+ = \epsilon_0^- = 0.001\,/\mathrm{s}$ and $F_d^+ = F_d^- = 6\,\mathrm{pN}$. Both the average elastic force $\langle F^+ \rangle(t)$ acting on both kinesins and the average elastic force $\langle F^- \rangle(t)$ experienced by the dynein increase monotonically with time t and approach the values predicted by the non-elastic model $F^{\pm}_{\mathrm{MKL}}$ (dashed and dotted horizontal lines) for large t. In contrast to the behavior displayed in Fig. 7(b) of the main text, no maxima are observed at intermediate times t because strain-induced unbinding events are strongly suppressed.

S4 Tug-of-war in the limit of small unbinding rates

In Fig. 6 of the main text we investigate the limiting case of small unbinding rates for all motors, and observe that the steady state forces approach the values predicted by the non-elastic MKL model. We now consider the time evolution of the elastic forces in the same limit for the tug-of-

war of two kinesins and one strong dynein. Inspection of Fig. S2 reveals that the time evolution of the limit system leads to a monotonous increase in the elastic forces generated during the tug-of-war. Thus, we conclude that antagonistic motor teams continuously step in their preferred direction of movement and that occasional backward steps do not lead to a maximum of $\langle F^{\pm}\rangle(t)$. Furthermore, in this limit the timescale for the force relaxation is about $t \simeq 1000\,\mathrm{ms}$, which is much longer than the corresponding timescale of approximately 300 ms for the case considered in the main text, see Fig. 7(b).

S5 Non-elastic model for (2+1) motors

The Müller-Klumpp-Lipowsky (MKL) model [5, 6, 7] provides a description of a tug-of-war between multiple antagonistic motors without elastic interactions. The model is based on a coarse-grained description of motor motion that does not include single steps taken by the motors. According to the MKL model, when two antagonistic motors are simultaneously bound to the filament they exert opposing forces onto each other through the cargo particle. The value of this interaction force, the cargo force F_{ca}, is determined by the following assumptions: As a result of the cargo force, both the plus and the minus motors reduce their velocities and the whole cargo-motor complex moves with a single velocity, the cargo velocity v_{ca}, determined by an instantaneous velocity matching of both motor teams [7, 1].

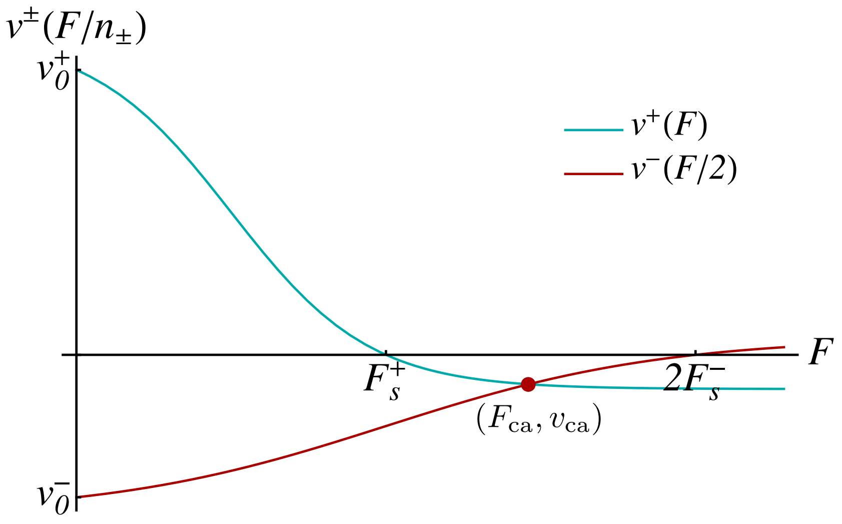

Figure S3: **Cargo force of the non-elastic MKL model:** Rescaled force-velocity relations for $n_- = 2$ minus and $n_+ = 1$ plus motors with identical stall forces $F_s^+ = F_s^-$. The cargo force F_{ca} of the MKL model is determined by the velocity matching condition $v^+(F_{\mathrm{ca}}/n_+) = v^-(F_{\mathrm{ca}}/n_-) = v_{\mathrm{ca}}$, where both motor teams move with the same velocity v_{ca} into the direction of the stronger motor team. The equal force sharing assumption then implies that at the intersection point $(F_{\mathrm{ca}}, v_{\mathrm{ca}})$ the force acting on both minus motors is given by $F^- = F_{\mathrm{ca}}/2$, whereas the single plus motor is subject to $F^+ = F_{\mathrm{ca}}$.

In the MKL model, the force acting on the motors is *assumed to be shared equally* by all motors of the same team [6, 7]. This equal force sharing condition implies that the force-velocity relation of the plus or minus motor team is given by the rescaled expression $v^{\pm}(F/n_{\pm})$, where F is the external force acting on the motors, and $F/n_{\pm}$ is the force acting on a single motor of the plus or minus motor team. The velocity matching condition of opposing motors can be visualized by plotting the rescaled force-velocity relations of a single plus and minus motor in the same (F, v)-diagram [1]. Fig. S3 displays the rescaled force-velocity relations of the motors for $n_{+} = 1$ plus and $n_{-} = 2$ minus motors simultaneously attached to the filament. The cargo force $F_{\mathrm{ca}}(n_{-} = 2, n_{+} = 1)$ and the cargo velocity $v_{\mathrm{ca}}(n_{-} = 2, n_{+} = 1)$ are then given by the coordinates at the intersection of both force-velocity relations. At the intersection point $(F_{\mathrm{ca}}, v_{\mathrm{ca}})$ the two attached minus motors share the cargo force equally, *i.e.*, $F^{-} = F_{\mathrm{ca}}/2$, while the single plus motor bears $F^{+} = F_{\mathrm{ca}}$. Note that the equal force sharing condition of the MKL model implies an additive force generation of motors by increasing number of motors. Thus the force value to stall a single motor in a given motor team increases additively with the number of attached motors in that team, *i.e.*, $F = n_{\pm}F_{s}^{\pm}$.

References

[1] Uçar MC, Lipowsky R. Tug-of-war between two elastically coupled molecular motors: a case study on force generation and force balance. Soft Matter. 2017;13:328–344.

[2] Mathematica. Version 9.0 ed. Champaign, Illinois: Wolfram Research, Inc.; 2012.

[3] Lindemann CB, Hunt AJ. Does axonemal dynein push, pull, or oscillate? Cell motility and the cytoskeleton. 2003;56(4):237–244.

[4] Oiwa K, Sakakibara H. Recent progress in dynein structure and mechanism. Curr Opin Cell Biol. 2005;17(1):98–103.

[5] Müller MJ, Klumpp S, Lipowsky R. Tug-of-war as a cooperative mechanism for bidirectional cargo transport by molecular motors. Proc Natl Acad Sci U S A. 2008;105(12):4609–4614.

[6] Müller MJ, Klumpp S, Lipowsky R. Motility states of molecular motors engaged in a stochastic tug-of-war. J Stat Phys. 2008;133(6):1059–1081.

[7] Lipowsky R, Beeg J, Dimova R, Klumpp S, Müller MJI. Cooperative behavior of molecular motors: cargo transport and traffic phenomena. Physica E. 2010;42(3):649–661.
